# Discriminative pattern discovery for the characterization of different network populations

**DOI:** 10.1093/bioinformatics/btad168

**Published:** 2023-04-06

**Authors:** Fabio Fassetti, Simona E Rombo, Cristina Serrao

**Affiliations:** DIMES, University of Calabria, Via Pietro Bucci, 41C, Rende, CS 87036, Italy; DMI, University of Palermo, Via Archirafi, 34, Palermo 90123, Italy; DIMES, University of Calabria, Via Pietro Bucci, 41C, Rende, CS 87036, Italy

## Abstract

**Motivation:**

An interesting problem is to study how gene co-expression varies in two different populations, associated with healthy and unhealthy individuals, respectively. To this aim, two important aspects should be taken into account: (i) in some cases, pairs/groups of genes show collaborative attitudes, emerging in the study of disorders and diseases; (ii) information coming from each single individual may be crucial to capture specific details, at the basis of complex cellular mechanisms; therefore, it is important avoiding to miss potentially powerful information, associated with the single samples.

**Results:**

Here, a novel approach is proposed, such that two different input populations are considered, and represented by two datasets of edge-labeled graphs. Each graph is associated to an individual, and the edge label is the co-expression value between the two genes associated to the nodes. Discriminative patterns among graphs belonging to different sample sets are searched for, based on a statistical notion of ‘relevance’ able to take into account important local similarities, and also collaborative effects, involving the co-expression among multiple genes. Four different gene expression datasets have been analyzed by the proposed approach, each associated to a different disease. An extensive set of experiments show that the extracted patterns significantly characterize important differences between healthy and unhealthy samples, both in the cooperation and in the biological functionality of the involved genes/proteins. Moreover, the provided analysis confirms some results already presented in the literature on genes with a central role for the considered diseases, still allowing to identify novel and useful insights on this aspect.

**Availability and implementation:**

The algorithm has been implemented using the Java programming language. The data underlying this article and the code are available at https://github.com/CriSe92/DiscriminativeSubgraphDiscovery.

## 1 Introduction

The analysis of gene expression data may contribute to unravel the complex mechanisms that influence the occurrence and course of disorders and diseases (Rung and Brazma 2013). An interesting problem in this context is to identify relevant factors, related to gene expression in different populations, able to characterize the unhealthy status with respect to the healthy case. As an example, one of these factors may be represented by the presence of genes that are significantly co-expressed in unhealthy individuals rather than in healthy ones, or vice versa.

It is well known that complex diseases are often characterized by collaborative effects involving multiple genes/proteins, also referring to the co-expression of different genes under specific conditions (Anastassiou 2007; Watkinson et al. 2008). Effective models to represent gene expression data in this context are the “co-expression networks,” where nodes are associated to genes and they are linked by edges if the corresponding genes are co-expressed. Usually, co-expression networks represent the input sample set globally (Liu et al. 2016; van Dam et al. 2018). However, it has been observed that the gene expression profiles often share local, rather than global, similarities (Roy et al. 2014). Therefore, in modeling a population of individuals through a single graph, some potentially powerful details on the co-expressions occurring in different samples may be left aside.

Here, we propose an approach based on the analysis of co-expression networks to identify interesting differences between two input sample sets, associated to healthy and unhealthy individuals, respectively. Two main points characterize the proposed approach:

1. A representation of gene co-expression data able to take into account local similarities, by turning the input gene expression dataset into a graph dataset, where there is a labeled graph for each sample.
2. The definition of a suitable notion of “discriminative patterns,” useful to capture the differences between the two input sample sets.

Other discriminative graph pattern mining approaches have been proposed previously. As an example, Yan et al. (2008) search for those graph patterns that occur with disproportionate frequency in some graphs versus others, and consider the bioassay records for anticancer screen tests with different cancer cell lines. However, in that case, patterns discriminate among different graphs, rather than between two different graphs populations as proposed here. Ting and Bailey (2006) introduce the notion of “minimal contrast subgraph pattern” between a single positive graph and a set of negative ones, for the comparison of chemical compounds. They are interested in finding edge sets in the positive graph which do not occur in the negative samples set. However, they do not take into account the pattern frequency, and related relevance, that is instead a key aspect here. Moreover, their approach cannot be easily extended to pairs of populations. “Synergy graph patterns” have been defined by Wang et al. (2015), referring to subgraphs such that the relationships among the nodes are highly inseparable. They apply a classification algorithm based on synergy graph patterns to real-life datasets, such as an AIDS antiviral screen chemical compounds dataset, and anticancer screen datasets. Similar to our approach, they consider only those graph patterns with discriminative powers much higher than all their subgraphs. However, their notion of discriminative power is defined differently than here. Most importantly, all mentioned approaches have been applied in contexts different than gene co-expression data, while that proposed here is specifically designed for the study of gene co-expression variation in healthy/unhealthy populations. Moreover, all approaches recalled above have mainly classification purposes, while our goal is to provide compact descriptors able to single out important functional differences between the two input datasets for further analysis.

In more detail, the approach proposed here considers all the complete information characterizing gene co-expression in each sample of the input sets. Therefore, it is able to capture also relevant collaborative effects occurring in single individuals and which emerge during the pattern extraction process. To this aim, the discriminative power of each pattern is measured based on a notion of information entropy, which takes into account both the pattern support and the co-expression levels of genes involved in the pattern, in one of the two datasets with respect to the other. The methodology has been validated on synthetic data and applied to analyze four different gene expression datasets, each associated to a different disease and containing the expression levels of genes for healthy and unhealthy samples, respectively. The considered diseases are “prostate cancer,” “pancreas cancer,” “gastric cancer,” and “psoriasis.” An extensive set of experiments has been performed on these datasets, at first with the aim of understanding to what extent the extracted patterns significantly characterize important differences between healthy and unhealthy samples. Then, further analysis of the obtained results has been provided, in order to identify novel and useful insights associated to the considered diseases.

In particular, enrichment analysis shows that for most of the retrieved patterns (over the 93% for three out of the four considered diseases) the intercepted genes are involved in common biological processes/functions, often relevant for the associated diseases. Moreover, protein–protein interaction (PPI) network analysis shows that some of the genes involved in the extracted patterns correspond to proteins that are “hubs” for human, i.e. they interact with a large number of other proteins to perform important functional tasks in the cell. On the other hand, both size and structure of patterns look different depending on the fact that they characterize healthy or unhealthy populations, respectively. Indeed, patterns characterizing unhealthy samples often present a simpler structure and smaller size than those associated to healthy populations, suggesting that passing from the healthy to the unhealthy status may be associated to the failure of some collaborative effects in the cell.

Finally, another interesting finding is that, for all considered datasets, a few genes co-occur in a large number of patterns. To this respect, the frequency of occurrence of single genes, as well as pairs, triples, and quadruples of them reveals the presence of “building blocks” recurrent inside the extracted patterns. By looking in more detail at such building blocks, it is possible to observe that they contain both genes which are known in the literature to be associated with the disease under consideration, and other genes, known to be implied instead in other diseases. This leads to the consideration that the proposed approach may be usefully applied also to discover novel putative associations between genes and diseases, as well as possible risk factors involving different diseases.

## 2 Methods

Let **DS** be a population of individuals such that, for each individual, the expression levels of the genes are known. **DS** can be represented by a set of tuples defined on a set of attributes, such that each tuple *t* is associated with an individual of **DS** and each attribute *a* is associated with a gene. The value *t*(*a*) which a tuple *t* assumes on the attribute *a* represents the expression level of the gene associated with *a* for the corresponding individual. For the sake of simplicity, in the following the symbol *t* will be used to denote indifferently the individual or its corresponding tuple in **DS** (the same holds for genes and attributes, respectively).

### 2.1 Characterizing pairs of genes

Given two genes *a*_1_ and *a*_2_, it is important quantifying to what extent they may be “associated,” based on their co-expression in the same individual. Let $X_{i}^{t},\;X_{j}^{t}$ be the random variables associated with $t(a_{i})$ and $t(a_{j})$, respectively. Consider the bivariate normal distribution $B_{ij}^{t}=N_{2}^{t}(\mu_{\mathrm{ij}},\Sigma_{\mathrm{ij}}^{t})$ having mean vector $\mu_{\mathrm{ij}}$ and co-variance matrix $\Sigma_{\mathrm{ij}}^{t}$, where:


$$\mu_{\mathrm{ij}}=\left(\begin{array}{c} \mu_{i}\\ \mu_{j} \end{array}\right),\Sigma_{\mathrm{ij}}^{t}=\left(\begin{array}{cc} \sigma_{i}^{2} & \rho_{ij}^{t}\sigma_{i}\sigma_{j}\\ \rho_{ij}^{t}\sigma_{i}\sigma_{j} & \sigma_{j}^{2} \end{array}\right).$$

In particular, *μ_i_* (*μ_j_*, respectively) is the mean value of the attribute *a_i_* (*a_j_*, respectively), *σ_i_* (*σ_j_*, respectively) is the standard deviation of the attribute *a_i_* (*a_j_*, respectively), and $\rho_{ij}^{t}$ is the correlation between $X_{i}^{t}$ and $X_{j}^{t}$.

The following two definitions are a revised version of those introduced by Fassetti et al. (2016).

Definition 1 (Strength). *The* strength *of the association between a_i_ and a_j_ for the individual t is the value of correlation* ${\tilde{\rho }}_{ij}^{t}$*such that the probability of observing the value* $t(a_{i})$*for* $X_{i}^{t}$*and the value* $t(a_{j})$*for* $X_{j}^{t}$*is maximum*.

Intuitively, the strength of the association between two genes quantifies the correlation between their values of expression. In order to estimate the statistical significance of the strength values, i.e. to measure the probability that a possible high value of correlation is not due by chance under the Null Hypothesis that it is implied by a certain value of expression, the notion of “relevance” is defined as follows.

Definition 2 (Relevance). *Let a_i_ and a_j_ be two genes. The relevance of the association between a_i_ and a_j_ for t is the minimum between the probability of observing a strength smaller than* ${\tilde{\rho }}_{ij}^{t}$*, given the expression level* $t(a_{i})$*, and the probability of observing a strength smaller than* ${\tilde{\rho }}_{ij}^{t}$*, given the expression level* $t(a_{j})$.

Details on the computation of strength and relevance are provided in Supplementary Section S1.1.

### 2.2 Characterizing a population

Let, **DS** be a population and *t* be an individual in **DS**. The individual *t* has a specific configuration of gene associations that can be characterized according to the following definition.

Definition 3 (WIGA-network). *A* Weighted Individual Gene Association Network (WIGA-network, *for short) is a weighted graph* (V,E,η)*such that:*

- *Each node* $v_{i}\in V$*represents a gene a_i_ of t*,
-
η:E→R *is a function associating each edge* $(v_{i},v_{j})\in E$*with a real number representing the strength of the association between the corresponding genes a_i_ and a_j_.*

In order to avoid the presence of edges corresponding to insignificant associations between genes, only those edges corresponding to pairs of genes such that the relevance of their association is larger than a value *τ_r_* fixed *a priori* (and equal to 0.9 in our experiments), are left on. We will refer to such “filtered” WIGA–networks in the following.

If **DS** consists of *m* individuals, a set **N** of *m* WIGA–networks results, such that the *i*th WIGA–network$N_{i}$ is associated with the *i*th individual of **DS**. All WIGA–networks are defined on the same set of nodes *V*, due to the fact that individuals in the same population have the same genes. The differences among the WIGA–networks in **N** are in their topologies and/or edge weights.

One of the main goals here is to characterize a population **DS** according to the most significant patterns that are recurrent in **N**. The following definitions are introduced to this aim.

Definition 4 (Pattern). *A* pattern P*of* ***N****is a connected graph (Vp, Ep) such that:*

-
Vp⊆V ,
- *there exists at least a* WIGA–network $N_{i}=(V,E_{i},\eta_{i})$ *in* ***N** such that* $E_{P}\subseteq E_{i}$ , i.e. P*occurs* or has a *match* in $N_{i}$.

Given a pattern P, for each WIGA–network in **N** at most a match of P may exist, due to the fact that all WIGA–networks are defined on the same set of nodes (i.e. the genes of the individuals in **DS**).

Definition 5 (Subpattern/Superpattern). *Let*, $P=(V_{P},E_{P}),\;P\prime =(V_{P\prime },E_{P\prime })$*be two patterns of* **N***.* P′*is a* subpattern *of* P*(*P*is a* super-pattern *of* P′*, respectively) if* $V_{P\prime }\subseteq V_{P}$*and* $E_{P\prime }\subseteq E_{P}$*. This is denoted by* P′≼P.

The same pattern may occur in different WIGA–networks, with the involved edges having different values of strength. The *value* of a match for a pattern in a WIGA–network is defined as follows.

Definition 6 (Value of a match). *Let* N=(V,E,η)*be a* WIGA–network*in* **N***and* $P=(V_{P},E_{P})$*be a pattern of* ***N****which occurs in* N*. The value* η(P,N)*of the match of* P*in* N*is defined as:* $\eta (P,N)=\frac{1}{\left|E_{P}\right|}\cdot \sum_{e\in E_{P}}\eta (e).$

It is expected that the most significant patterns occur with high value matches on a large sample of the considered population. Given a pattern P of **N**, its *incidence* is defined below.

Definition 7 (Incidence of a pattern). The *incidence* of P on **N** is defined as: $s(P,N)=\sum_{N\in N}\eta (P,N).$

Property 1. *The incidence of* P*on* **N***is upper bounded by its support, i.e., the number of* WIGA–networks*where* P*occurs.*

This latter property immediately follows from the fact that the value of a pattern match in a WIGA–network ranges in [0,1].

The following definition allows to understand to what extent a pattern P “characterizes” the considered population **DS**, given that it occurs at least on a fixed percentage of the associated networks.

Definition 8 (Incidence of a pattern at x%). *Let* P*be a pattern of* ***N****. If the support of* P*in* N*is less than the* x%*of* N*, then the incidence of* P*at the* x%*of* N*is null. Otherwise, it is defined as:* $\hat{s}(P,N)=\sum_{N\in \hat{N}}\eta (P,N)\;$*where* $\hat{N}$*are the first* x%*WIGA-networks in* N*where* P*occurs, sorted in decreasing order with respect to the corresponding values of* P*matche*s.

From this latter definition, it follows that **compact descriptors** may be provided **for a population**, made of all its patterns having non-null incidence at a given percentage. In the following, if not differently specified, we assume that a percentage *x* has been fixed *a priori*.

### 2.3 Discriminating different (sub)populations

Let, $DS_{1}$ and $DS_{2}$ be two subpopulations of **DS**, such that the partition is based on some properties of the samples independent from the gene expression levels. Here we consider “healthy versus unhealthy” individuals, for a given disease. The proposed approach aims to investigate if, and to what extent, “macroscopic” differences may be identified in the co-expression of genes corresponding to individuals in the two different partitions. Given that in the previous section, it has been explained how a population can be characterized by a compact set of patterns, the attention now turns on identifying those patterns which characterize one subpopulation, with reference to the other one. Indeed, such patterns may help to *discriminate* between the two subpopulations.

Let, $N_{1}$ and $N_{2}$ be the two sets of WIGA–networks associated to $DS_{1}$ and $DS_{2}$, respectively, such that $N=N_{1}\cup N_{2}$. In order to measure the “discriminative power” of a pattern, the notion of information gain (Mitchell 1997) is considered and adapted to this context. The aim is to measure the change in information entropy (Gray 2011) induced by the pattern on the population **DS**, with regards to its subpopulations $DS_{1}$ and $DS_{2}$.

Definition 9 (Information entropy). The *information entropy* H(N) may be defined as: $H(N)=–\frac{\left|N_{1}\right|}{\left|N\right|}\text{log}\;\frac{\left|N_{1}\right|}{\left|N\right|}–\frac{\left|N_{2}\right|}{\left|N\right|}\text{log}\;\frac{\left|N_{2}\right|}{\left|N\right|}.$

Suppose that a pattern P of **DS** partitions **N** in two groups: $N^{P}$, i.e. the subset of WIGA–networks in **N** that contains P, and $N^{\bar{P}}$, i.e. the WIGA–networks which do not contain P.

Definition 10 (Information entropy given a pattern). *Let* P*be a pattern. The information entropy given a pattern* H(N|P)*is:where* $q=\frac{\hat{s}(P,N_{1})+\hat{s}(P,N_{2})}{\left|N\right|}$, *and:where* $q_{1}=\frac{\hat{s}(P,N_{1})}{\hat{s}(P,N_{1})+\hat{s}(P,N_{2})},\;$$q_{2}=\frac{\left|N_{1}|–\hat{s}(P,N_{1}\right)}{\left|N_{1}|–\hat{s}(P,N_{1})+|N_{2}|–\hat{s}(P,N_{2}\right)}.$

It is worth pointing out that Definition 10 is not symmetric. Indeed it aims to highlight those patterns which characterize $DS_{1}$ but not $DS_{2}$, due to the fact that they have induced a variation of entropy according to the above *q*_1_ and *q*_2_. The vice versa can be easily obtained by switching $N_{1}$ and $N_{2}$ in the definition.

Definition 11 (Discriminative power). *The* discriminative power *of the pattern* P*, denoted by* pow(P)*, is the gain in entropy:* pow(P)=H(N)–H(N|P).

$$H(N|P)=H(N^{P})\cdot q+H(N^{\bar{P}})\cdot \left(1–q\right),\;$$

$$H(N^{P})=–q_{1}\;\text{log}\;q_{1}–(1–q_{1})\;\text{log}(1–q_{1}),\;$$

$$H(N^{\bar{P}})=–q_{2}\;\text{log}\;q_{2}–(1–q_{2})\;\text{log}(1–q_{2}),\;$$

In Supplementary Section S1.2, it is discussed how to determine an upper bound for the discriminative power, which will be useful to prune the search space in the discovery process.

Definition 12 (Discriminative pattern). *A pattern* P*is discriminative if, for each pattern* P′≼P*, one of the two following conditions holds: (1)* pow(P)>pow(P′)*, (2)* pow(P)=pow(P′)*and* $\hat{s}(P)>\hat{s}(P\prime )$.

Therefore, a discriminative pattern has discriminative power larger than that of all its subpatterns. However, we are interested in discovering patterns that are also *maximal*, according to the following definition.

Definition 13 (Maximal discriminative pattern). *A discriminative pattern* P*is maximal if there is not any other discriminative pattern* P′*such that* P≼P′.

### 2.4 Algorithms

Given two (sub)populations $DS_{1}$ and $DS_{2}$, the main goal here is to extract the most representative discriminative patterns between them (The algorithms described here are improved versions of those in Fassetti et al. 2016.). To this aim, the function TopKPatterns for the extraction of maximal discriminative patterns is considered.

TopKPatterns takes in input two sets of WIGA–networks$N_{1}$ and $N_{2}$, associated to $DS_{1}$ and $DS_{2}$, respectively. Its output is the set of top-*k* discriminative patterns which distinguish samples in $DS_{1}$ from those in $DS_{2}$ (the vice versa may be obtained analogously), sorted with references to their discriminative power. The function works as follows. Unidimensional patterns are generated at the beginning, by taking each edge represented in at least one network in $N_{1}$ (getEdges). Each of these patterns, P is considered for possible extension by the recursive function PatternMine, in order to generate larger and potentially more interesting ones. The final set, containing all (possible) extensions of the initial patterns is finally pruned (Prune) by eliminating residual redundancy, according to Definition 13, and the top-*k* patterns sorted according to their discriminative power (TopK) are selected.

**Function**PatternMine(P)

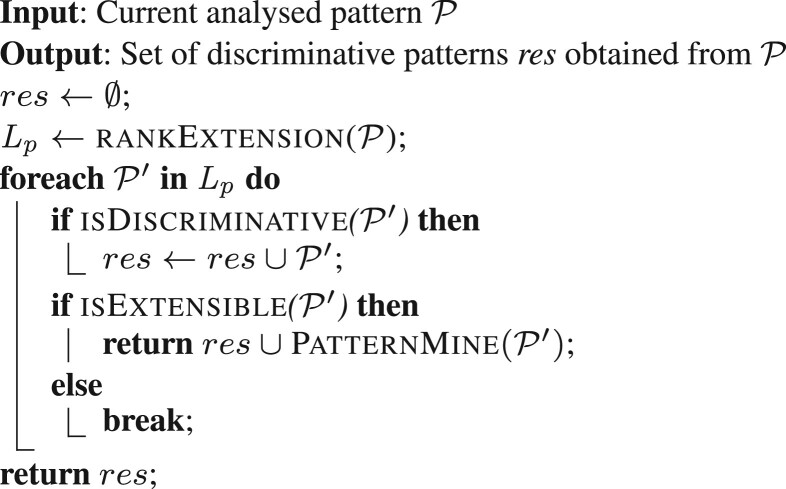

The main core of TopKPatterns is the function PatternMine, based on a *depth first* strategy applied to navigate and prune the search space, consisting of the connected subgraphs of the input networks. PatternMine receives in input a pattern P. At the beginning, the pattern result set *res* is set equal to the empty set, and the function RankExtension is called to generate the list *L_p_* of all possible extensions of P. A possible extension is a pattern P′ obtained from P by adding a new edge that (i) connects two nodes already in P, or (ii) involves a new external node. *L_p_* is sorted in decreasing order, with regards to the patterns *incidence*. Then, for each pattern P′ in *L_p_*, the function isDiscriminative checks if it is *discriminative* according to Definition 12. In affirmative case, P′ is inserted in the result-set *res*.

Each pattern P′ in *L_p_* has also to be checked for possible further expansion. To this aim, the upper bound discussed in Supplementary Section S1.2 has a key role. Indeed, it provides a measure of the largest possible discriminative power that can be obtained by extending it. Therefore, those patterns in *L_p_* whose upper bound is lower than the discriminative power of the pattern P from which they have been generated, will not be extended any more. Furthermore, as the upper bound grows together with the *incidence* and *L_p_* is sorted with respect to this measure, if a pattern cannot be extended then all the other ones that follow it in *L_p_* can be safely pruned.

The function isExtensible executes the upper bound check on each pattern P′ in *L_p_* and, if successful, the patternMine function is recursively called until one of the following two conditions is verified: (i) the pattern cannot be extended anymore, (ii) the pattern has reached a maximum size eventually fixed *a priori*.

**Function** TopKPatterns $\left(N_{1},N_{2},k\right)$

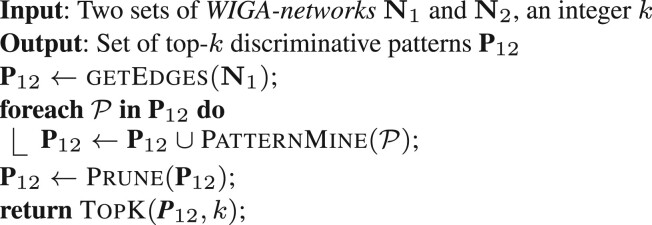

## 3 Results

The proposed approach has been applied to both synthetic and real data. In particular, Supplementary Section S2 describes experiments performed on a simulation scenario aiming at evaluating the robustness of the proposed approach. Real data have been retrieved from four different datasets of the gene expression omnibus public functional genomics data repository (Edgar et al. 2002). Each dataset refers to a disease and contains the expression levels of a certain number of genes for two different samples, associated to healthy and unhealthy individuals, respectively. In the following, we refer to each dataset by the name of the corresponding disease, i.e. “prostate cancer” (*GSE*68907), “pancreas cancer” (*GSE*15471), “gastric cancer” (*GSE*65801), and “psoriasis” (*GSE*13355). Datasets features are summarized in Supplementary Table S4.

Supplementary Table S5 shows a summary of some statistics on the discriminative patterns extracted at 20% of the considered populations, in both cases of “healthy versus unhealthy” (healthy, for short) and the vice versa (unhealthy), for each dataset. The number of patterns is between 286 and 654, while their size is between 2 and 11 genes. It is worth remarking that the proposed approach is “ asymmetric,” in that it aims at building a compact set of patterns which characterize one subpopulation, with reference to the other one. In particular, the first subpopulation “guides” the extraction process, thus that the patterns resulting in the highest discriminative power are those most representative of that population in contrast to the second one. This allows to accomplish the final goal of discovering which co-expression relationships characterize each of the two subpopulations in comparison.

The following subsections illustrate the different types of analysis performed on the considered datasets.

### 3.1 Functional enrichment analysis

For each considered dataset, and for each of the healthy and unhealthy cases, a “global view” of the extracted discriminative patterns has been generated as follows (see also Supplementary Fig. S1). All genes taking part in at least one pattern of the result set are considered, and an edge is put between a pair of genes if they are connected in at least one of these patterns. Edge weights are also considered, scoring the number of patterns where that edge occurs. A global view may include different connected components.

Functional enrichment analysis has been performed on all sets of genes intercepted by patterns included into the same connected components, to infer their possible association with disease phenotypes. Only connected components with size larger than 4 have been considered. The analysis has been performed from the home page of the gene ontology (GO) website, by the GO service that connects to the analysis tool from the PANTHER Classification System (Mi et al. 2013).

Gene sets with a *P*-value<.05 have been considered significantly enriched, with references to each of the three GO vocabularies, i.e. “biological process,” “molecular function,” and “cellular component.” As shown in Table 1, the percentage of patterns involving sets of genes significantly enriched is very large (over 93%) for pancreas, gastric, and prostate cancer and for almost all three GO vocabularies. For psoriasis, the obtained connected components are smaller with only two having size larger than 4. Results obtained from the functional enriched analysis are discussed below.

**Table 1.** Percentage of patterns involving sets of genes significantly enriched, for each GO vocabulary and dataset, and both Healthy (H) and Unhealthy (U). When connected components have size lower than 4, the enrichment analysis has not been performed (denoted by – in the table).

Prostate cancer		Pancreas cancer		Gastric cancer		Psoriasis	
H	U	H	U	H	U	H	U
Biological process							
1	0.93	0	0.97	0.96	0.97	0.14	–
Molecular function							
1	0.93	1	0.97	0.96	0.97	0.26	–
Cellular component							
1	0.93	0	0.97	0.96	0	0.34	–

#### 3.1.1 Prostate cancer

For this dataset, the largest number of GO annotations has been returned from the enrichment analysis. In particular, the healthy case is characterized by significant annotations for biological process, which include translation, peptide biosynthetic and metabolic processes, amide biosynthetic process. The unhealthy case also presents a large number of significant annotations such as response to stimulus, multicellular organismal process, and development, response to stimulus, cytoplasmic translation, peptide, and amide biosynthetic process, mostly confirming other previous findings on prostate cancer (see, e.g. Sun et al. 2019).

#### 3.1.2 Pancreas cancer

In this case, patterns discriminating the healthy population do not include sets of genes involved in significant biological processes. On the other hand, the related main molecular functions involve serine-type endopeptidase, peptidase, and hydrolase activity, as well as endopeptidase, peptidase, and hydrolase activity. Patterns discriminating the unhealthy case intercept genes characterized by antimicrobial humoral response and proteolysis, accordingly to Bharmal et al. (2017), who have found that pancreatic hormones exhibit a differential effect on the pancreatic proteolytic enzymes. Genes in the patterns result significantly enriched in molecular functions such as hydrolase, catalytic, lipase, and peptidase activity.

#### 3.1.3 Gastric cancer

Patterns characterizing the healthy case intercept sets of genes significantly enriched with terms related to sensory perception (of smell) and nervous system process, which has been identified also by Wang et al. (2021) as possibly implied in this type of cancer. For the unhealthy case, G protein-coupled receptor signaling pathway and detection of chemical stimulus involved in sensory perception have been found, accordingly to both Ge et al. (2019) and Tian et al. (2019).

#### 3.1.4 Psoriasis

The healthy population in Psoriasis is discriminated from the unhealthy one by patterns including genes involved in lipid, sterol, cholesterol, and alcohol metabolic processes. This confirms some recent findings by Nowowiejska et al. (2021), showing that psoriatic patients suffer frequently from obesity, dyslipidemia, and liver disease, also due to the fact that lipid expression and metabolism disorders are often present in such patients. Moreover, the associated proteins result to be involved in oxidoreductase activity, binding, acyltransferase, and catalytic activity.

### 3.2 Shape of the resulting patterns

Most of the generated patterns involve the co-occurrence of groups of genes linked in non-linear structures and pattern shapes seem to vary across to the different populations, as discussed in detail below. Supplementary Table S6 shows the top-12 patterns scoring the highest commonness values for prostate cancer (plots for the other datasets are analogous and they are not shown).

#### 3.2.1 Prostate cancer

Patterns characterizing the healthy population include either chains or more complex structures: some genes exist that keep the pattern joined or there are triplets of genes organized to form triangles, stating that a kind of inter-dependency exists in their expression. When moving to the unhealthy case, simpler structures emerge, and most patterns with high incidence are just chains formed by two or more genes.

#### 3.2.2 Pancreas cancer

Patterns getting higher incidence have all complex structures, in both healthy and unhealthy populations. The ones describing the healthy samples are based on a very small set of genes, which are variously combined; these genes result to be the building blocks characterizing the interactions in healthy samples. Some of them appear also in the patterns describing unhealthy; however, most of their interactions are different in the latter case, as if the pathological status implies both a *rewiring* among the genes, and the appearance or disappearance of interactions.

#### 3.2.3 Gastric cancer

Although patterns with up to six nodes have been extracted, the ones at the top of the ranking contain three or four nodes, mostly arranged to form chains or triangles. However, although no important differences may be highlighted in the pattern structures between healthy and unhealthy cases, we may point out that the genes involved in the two cases are completely different.

#### 3.2.4 Psoriasis

Here most extracted patterns are pairs. This is always true for the healthy population, whereas for the unhealthy one there are also a few larger patterns. Although pattern shapes and their sizes are different than in the previously analyzed datasets, we highlight a similarity with the prostate cancer case, in the fact that patterns in unhealthy present a simpler structure than in the healthy case.

### 3.3 Frequency of occurrence analysis

Another perspective of analysis is related to the fact that, for each considered dataset, a few genes occur in a large number of patterns. To this respect, the frequency of occurrence of single genes, as well as pairs, triples, and quadruples of them in the result-set has been analyzed (see Supplementary Tables S7–S10), and the DisGeNET discovery platform (Bauer-Mehren et al. 2010; Piñero et al. 2019) has been used to study the implication of such frequent genes in diseases.

#### 3.3.1 Prostate cancer

Healthy population for this dataset is characterized by patterns involving genes that code for ribosomal proteins, often combined with each other and with the gene *SRP*14. The unhealthy population presents apparently a more variegated set of genes frequently occurring in the extracted discriminative patterns. The gene *SPINK*2 is the most frequent one. Its encoded protein acts as a trypsin and acrosin inhibitor in the genital tract, and it is localized in the spermatozoa, being also associated with the progression of lymphomas Fagerberg et al. (2014). Looking at the co-occurrence of 3/4 genes in the unhealthy case, it results that the most frequent triples/quadruples involve the gene *AMELX* combined with genes coding for ribosomal proteins. This confirms previous studies, where an altered expression of *AMELX* was found to be associated to prostate cancer [see, e.g. research by Hong et al. (2015)]. Moreover, these results suggest that the co-expression of *AMELX* with genes coding for ribosomal proteins could play some roles in the occurrence and/or progress of the considered disease. Interestingly, many of the patterns characterizing the unhealthy population involve the two genes *HSPB*1 and POU3F1, often co-occurring with genes coding for ribosomal proteins (i.e. showing the same behavior of *AMELX*). This is another novel result, which may deserve further investigation. Indeed, such two genes are known to be associated with other types of cancer, such as Liver Carcinoma Cheng et al. (2015) and Lung Cancer Ubhi and Price (2005).

#### 3.3.2 Pancreas cancer

The main players in this case are the genes associated to the two enzymes CELA3A and CELA2B, which are characteristic of both the healthy and unhealthy populations. CELA3A is associated to exocrine pancreatic insufficiency (Vanga et al. 2018) and diabetes mellitus (Riceman et al. 2019), whereas CELA2B is determinant of blood pressure and body mass index (Giri et al. 2019). What is different in the patterns between healthy and unhealthy is the set of other genes with which CELA3A and CELA2B co-occur. Indeed, in the healthy case they are often paired with *SYNC*, encoding a protein highly expressed in skeletal and cardiac muscle, where it has a structural role. In the unhealthy population they co-occur with REG1A, REG1B, *CPB*1, and REG3A, all involved in diabetes mellitus and malignant neoplasms. This confirms what already observed also in Section 3.2: a few genes such as CELA3A and CELA2B have an important role in Pancreas Cancer, and in particular their interplay with other genes change when the disease arises and develops.

#### 3.3.3 Gastric cancer

For this disease the healthy population is characterized by the co-occurrence of both genes, such as *RTL*1, *PSG*3, *F*9, and non-coding RNA, such as *SERPINA*13 and *ADAM*6. The case “unhealthy versus healthy” presents the gene *BTG*4, already known to be associated with Gastric Cancer (Dong et al. 2009) as well as to Colorectal and Breast Carcinoma (Mori et al. 2011), often co-occurring with *LGALS*13 and *IFLTD*1, associated with Neoplastic Processes (Gilson et al. 2017) and respiratory tracts Neoplasms (Manenti et al. 2004), Cardiovascular Diseases (El Rouby et al. 2019), respectively. These latter co-occurrences may suggest possible risk factors for patients affected by Gastric Cancer to contract other types of Neoplasms, as well as Cardiovascular Diseases. Moreover, as in the previous analyzed cases, our analysis induces the hypothesis of new gene-disease associations for the considered disease.

#### 3.3.4 Psoriasis

The healthy case is characterized by the frequent co-occurrence of genes encoding different keratines (*KRT*35, *KRTAP*1-3, *KRTAP*3-3, *KRTAP*4-1, and *KRTAP*4-3). In the unhealthy case, as already explained in Section 3.2, the structure of patterns is not much complex and patterns are very diversified in the genes they involve. However, also in this case interesting examples of co-occurrences may be found, often involving genes known to be associated with Psoriasis, such as *QTRT*1 (Baurecht et al. 2015), as well as genes related to other diseases, e.g. *ERF*, implied in complex craniosynostosis (Twigg et al. 2013), and *ISOC*2, biomarker of Osteoarthrosis Deformans (Ruiz-Romero et al. 2009).

### 3.4 Hub gene identification via PPI network analysis

A further analysis has been performed to investigate if genes involved in the extracted patterns code for proteins, which may be considered “hubs” in the human PPI network, built by downloading data from IntACT (Orchard et al. 2014). It is well known that the degree distribution of PPI networks follows a power law, with many nodes having a low degree and few highly connected ones. Accordingly to Cui et al. (2020), hubs are nodes with degree at least 10.

A first observation is that the number of genes in the patterns which code for hub proteins is significantly larger for prostate cancer and psoriasis than for the other datasets, as evident from Supplementary Table S11. A second observation is that, in some of the considered populations, hubs corresponding to genes in the patterns have a degree considerably larger than 10 (e.g. 349 for prostate cancer). Supplementary Table S12 shows the top-25 hub proteins and their corresponding degrees, also discussed in detail below.

#### 3.4.1 Prostate cancer

For the healthy population, the hub with the largest degree (87) is *RPS*6, involved in the catalysis of protein synthesis and contributing to the control of cell growth and proliferation, through the selective translation of particular classes of mRNA (O’Leary et al. 2016). For the unhealthy case, the top-25 hubs range from 349 to 74 connections with other proteins in the network. Among them, there is *HSPB*1, a member of the small heat shock protein family of proteins which, in response to environmental stress, translocates from the cytoplasm to the nucleus and functions as a molecular chaperone that promotes the correct folding of other proteins (O’Leary et al. 2016). It has been proved that the expression of this gene is correlated also with poor clinical outcomes in multiple human cancers, and the encoded protein may promote cancer cell proliferation and metastasis (Ajalyakeen et al. 2020; Drexler et al. 2020). As for the hub RPS4X, it has been proven that dysregulation of *RP* expression occurs in a variety of human diseases, notably in many cancers (O’Leary et al. 2016), and altered expression of some RPs correlates with different tumor phenotypes and patient survival (Dolezal et al. 2018), including the prostatic one.

#### 3.4.2 Pancreas cancer

Patterns characterizing the healthy case come from the combination of only 10 genes, 6 out of which are also in the PPI network and present a small number of interactions. As for the unhealthy population, 27 out of the 38 genes involved in the patterns have a correspondence in the PPI network, where the highest degrees are scored by *FHL*1 and *KRT*20. *FHL*1 provides instructions for making three versions (isoforms) of a protein that plays an important role in muscles used for movement (skeletal muscles) and in the heart (cardiac muscle). *KRT*20 codes for a protein which is a member of the keratin family, the intermediate filament proteins responsible for the structural integrity of epithelial cells.

#### 3.4.3 Gastric cancer

Only a few genes in the patterns characterizing both the healthy and the unhealthy subpopulations code for proteins mapped in the considered PPI network. Among them, *PAX*2 belongs to the family of Paired-Box Containing Genes, which plays important roles in the development and proliferation of multiple cell lines, development of organs, and development and organization of the central nervous system (Mansouri and Gruss 2013). The corresponding transcription factor is important in the regionalized embryological development of the central nervous system, and it is believed to be a target of transcriptional suppression by the tumor suppressor gene *WT*1. Another hub is *IRS*4, a cytoplasmic protein that contains many potential tyrosine and serine/threonine phosphorylation sites. It characterizes the unhealthy case and interacts with other 103 proteins in the PPI network.

#### 3.4.4 Psoriasis

Differently than in previous cases, for Psoriasis most genes involved in patterns code for proteins mapped in the PPI network. There are 552 of such genes for the healthy and 595 for the unhealthy cases, respectively. Moreover, the degree of the intercepted hubs is often high, indeed the 43.84% and the 36.30% of proteins for healthy and unhealthy, respectively, have a degree larger than 10. The top hubs are *RELA*, encoding a transcription factor, for healthy, and *AGO*1, required for RNA-mediated gene silencing, for unhealthy.

### 3.5 Comparison against a standard approach

In Supplementary Section S3.5 further experiments comparing the proposed approach against a standard one, that identifies differentially expressed genes without considering co-expression among them, are presented. The main result is that, most of the genes involved in the best scoring discriminative patterns returned by the proposed approach and discussed in the previous paragraphs, would not have been detected by the standard approach, thus confirming the importance of taking into account collaborative effects, at the basis of the proposed approach.

## 4 Conclusion

An approach for the extraction of graph patterns useful to discriminate two different populations has been proposed and validated on gene expression data. Results show that, by the analysis of the extracted patterns, it is possible to identify significant differences between healthy and unhealthy samples, and also to investigate on the role of genes and cellular components in the occurrence and progress of diseases.

Interesting issues still remain open, e.g. the extension of the proposed approach to multiple datasets. This will require to extend the notions of information entropy and discriminative power introduced in Section 2.3, to account for the relevant differences among *n* sets of graphs. The goal will be to search for those graph patterns which characterize each set, with respect to the others *n−*1. We plan to study this problem in the future, referring to some application contexts where discriminating among different stages, associated to different populations, may be significant. Examples of that are datasets associated to patients at different states of a given disease, or to stem cells differentiation.

## Supplementary Material

btad168_Supplementary_Data
