# Supplementary material for: Discriminative pattern discovery for the characterization of different network populations
Source: Bioinformatics. 2023 Apr 6;39(4):btad168. doi: 10.1093/bioinformatics/btad168 (PMC10121338; doi:10.1093/bioinformatics/btad168)
Supplement: btad168_Supplementary_Data [file btad168_supplementary_data.pdf]

Supplementary Material for: Discriminative Pattern Discovery for the Characterization of Different Network Populations

Fabio Fassetti[*] Simona E. Rombo[†] Cristina Serrao[*]

1 Technical details of the approach

The proposed approach is based on two key notions, i.e., *Strength* and *Relevance*. In this section we provide first some technical insights aimed at clarifying how such measures have been defined and computed. Then, we show the listing corresponding to the core function of the proposed approach.

1.1 SR-Network weights computation

Let **DS** be a population, associated to a dataset as described in section 2 of the main manuscript, t be an individual in **DS** and a be a gene expressed by t. Each value $t(a)$, which represents the expression level of gene a in t, can be normalized with respect to the mean and the standard deviation of the other values for a in the same population, according to the z-score notion, that is: $\hat{t} = \frac{t(a)-\mu}{\sigma}$.

In the following, we deal with such normalized values and discuss in details how the values of *Strength* and *Relevance* have been computed for a given pair of genes a_i and a_j.

It is worth pointing out also that, the generation of WIGA-networks from gene expression data proposed here, depends on two thresholds, namely τ_s, for the strength, and τ_r, for the relevance. The strength is a measure of correlation, therefore we have considered the standard approach in statistical analysis according to which values of correlation up to 0.7 are significant, and we have set $\tau_s = 0.7$ through our experiments. As for the relevance, we note that it is a probability measure, so higher τ_r more probable the detected correlation. For the experiments discussed here, we have fixed $\tau_r = 0.9$.

Strength computation Let $\hat{t}_i$ and $\hat{t}_j$ be the z-score values of $t(a_i)$ and $t(a_j)$, and $\widehat{X}_i^t$, $\widehat{X}_j^t$ be the random variables associated with $\hat{t}_i$ and $\hat{t}_j$, respectively. Consider the bivariate normal distribution, which is usually assumed for gene-expression data, with components $\widehat{X}_i^t$ and $\widehat{X}_j^t$, mean vector $\widehat{\boldsymbol{\mu}}$ and covariance matrix $\widehat{\boldsymbol{\Sigma}}$, where:

$$\widehat{\boldsymbol{\mu}}_{\boldsymbol{ij}} = \begin{pmatrix} 0 \\ 0 \end{pmatrix}, \widehat{\boldsymbol{\Sigma}}_{\boldsymbol{ij}}^{\boldsymbol{t}} = \begin{pmatrix} 1 & \rho_{t_i t_j} \\ \rho_{t_i t_j} & 1 \end{pmatrix}.$$

The bivariate normal distribution can be written as:

$$f(x, y, \rho_{t_i t_j}) = \frac{1}{2\pi\sqrt{1-{\rho_{t_i t_j}}^2}} e^{-\frac{1}{2\left(1-{\rho_{t_i t_j}}^2\right)}\left(x^2+y^2-2\rho_{t_i t_j} xy\right)}.$$

The strength between a_i and a_j for t is the value $\tilde{\rho}_{t_i t_j}$ of $\rho_{t_i t_j}$ such that the value of f in the point $(\hat{t}_i, \hat{t}_j, \rho_{t_i t_j})$ is maximum; in formula:

$$\tilde{\rho}_{t_i t_j} = \arg\max_{\rho_{t_i t_j}} f(\hat{t}_i, \hat{t}_j, \rho_{t_i t_j}).$$

To evaluate the maximum of f we move to the logarithm as the logarithmic function is a continuous monotone increasing one:

[*]DIMES, University of Calabria, Rende (CS), Italy
[†]DMI, University of Palermo, Palermo, Italy

$$\log(f(x,y,\rho)) =$$
$$= \log\left(\frac{1}{2\pi\sqrt{1-\rho^2}}\right) + \left(-\frac{1}{2(1-\rho^2)}(x^2+y^2-2\rho xy)\right) =$$
$$= -\log(2\pi) - \frac{1}{2}\log\left(1-\rho^2\right) - \frac{1}{2}\left(\frac{x^2+y^2-2\rho xy}{1-\rho^2}\right)$$

Moreover, as adding a constant to a function does not alter the argument of the maximum, we add $\log(2\pi)$ and consider the maximum of

$$g(x,y,\rho) = -\frac{1}{2}\log\left(1-\rho^2\right) - \frac{1}{2}\left(\frac{x^2+y^2-2\rho xy}{1-\rho^2}\right).$$

Since we aim at finding the maximum of g as a function of ρ, we consider the partial derivative of g w.r.t. ρ:

$$\frac{\partial g}{\partial \rho} = \frac{\rho}{1-\rho^2} + \frac{(xy)(1-\rho^2) - (x^2+y^2-2\rho xy)\rho}{(1-\rho^2)^2}$$

and look for the stationary points by calculating the values of ρ where $\frac{\partial g}{\partial \rho} = 0$.

$$\frac{\rho}{1-\rho^2} + \frac{(xy)(1-\rho^2) - (x^2+y^2-2\rho xy)\rho}{(1-\rho^2)^2} = 0 \Longrightarrow$$
$$\rho^3 - \rho^2 xy + \rho(x^2+y^2-1) - xy = 0 \qquad (1)$$

Therefore, we get a cubic equation. By setting:

$$p = x^2+y^2-1-\frac{x^2y^2}{3}$$
$$q = -xy + \frac{xy(x^2+y^2-1)}{3} - \frac{2x^3y^3}{27}$$
$$\Delta = \frac{q^2}{4} + \frac{p^3}{27},$$

we obtain that the solutions of (1) depend on the sign of Δ. Particularly, two possible cases may occur:

$\Delta \geqslant 0$: We have just one real solution that, as can be easily verified, corresponds to a maximum:

$$\rho = \frac{xy}{3} + \sqrt[3]{-\frac{q}{2}+\sqrt{\Delta}} + \sqrt[3]{-\frac{q}{2}-\sqrt{\Delta}}$$

$\Delta < 0$: We should compute the square root of a negative number. This task has a solution in the set of complex numbers. Let define: $z_1 = -\frac{q}{2} + i\sqrt{-\Delta}$ and: $z_2 = -\frac{q}{2} - i\sqrt{-\Delta}$ Note that $z_1, z_2 \in \mathbb{C}$ and $z_2 = \overline{z_1}$. It follows that the solution of (1) can be written as: $\rho = \frac{xy}{3} + \sqrt[3]{z_1} + \sqrt[3]{z_2}$. As there are three complex roots, there are three values for rho that are solution of (1):

$$\rho_k = \frac{xy}{3} + \sqrt[3]{|z_1|e^{i\frac{\vartheta+2k\pi}{3}}} + \sqrt[3]{|z_2|e^{i\frac{-\vartheta+2k\pi}{3}}}$$

with $k = 0,1,2$. It follows that there are three solutions in $\mathbb{R}$:

$$\rho_1 = \frac{xy}{3}cos\left(\frac{\vartheta}{3}\right),$$
$$\rho_2 = \frac{xy}{3}cos\left(\frac{\vartheta+2\pi}{3}\right),$$
$$\rho_3 = \frac{xy}{3}cos\left(\frac{\vartheta+4\pi}{3}\right).$$

However, not all the solutions we have found are valid stationary points for g, because they are not in the function's domain. Therefore, among the valid values of ρ (i.e. $-1 \leq \rho \leq 1$), we have to choose only the one that maximize g.

Relevance computation Consider again the normalized expression values $\hat{t}_i$ and $\hat{t}_j$ and let ρ^* be the strength we can associated to such values. We are interested in evaluating the probability of observing a strength smaller than the observed one. We refer to such a probability as P_i^- and P_j^-, respectively. Then, the relevance between a_i and a_j for t is:

$$\min(P_i^-, P_j^-) = 1 - \max(P_i^-, P_j^-),$$

where P_i^- and P_j^- can be rewritten as:

$$P_i^- = 1 - Pr(\rho \geq \rho^*|\hat{t}_i) = 1 - P_i^+$$
$$P_j^- = 1 - Pr(\rho \geq \rho^*|\hat{t}_j) = 1 - P_j^+.$$

In order to evaluate the relevance, we can compute the probability P_i^+ (P_j^+, respectively) of observing a value of $\hat{t}_i$ ($\hat{t}_j$, respectively) such that the strength of a_i and a_j for t is greater than ρ^*, by keeping $\hat{t}_i$ ($\hat{t}_j$, respectively) fixed.

Consider Equation (1) again. By solving it with respect to x (y, respectively) and keeping ρ and y (x, respectively) fixed, we can determinate two points x', x'' such that the strength of a_i and a_j for t is greater that ρ^* for any $x' \leq \hat{t}_i \leq x''$. Particularly, given values ρ_0 and y_0, with $0 < \rho_0 \leq 1$, we aim at finding the values of x such that the value of ρ solution of Equation (1) is larger than ρ_0; the same line of reasoning can be followed to find a value for y such that the value of ρ solution of Equation (1) is larger than ρ_0, keeping x_0 fixed.
More formally, the following theorem holds:

Theorem 1 *Let ρ_0, x_0 and y_0 with $0 < \rho_0 \leq 1$ be such that Equation* (1) *holds with this input. Let, also, x' and x'' be the solutions of Equation* (1)*, solved w.r.t. x, by setting $\rho = \rho_0$ and $y = y_0$. For any $x' \leq x \leq x''$, the value of ρ such that Equation* (1) *holds is greater than ρ_0.*

Proof First of all note that Equation (1) is a quadratic equation w.r.t. to x then it cannot admit more than two solutions. Since ρ_0 is the solution of the equation when $y = y_0$, Equation (1) admits for sure real solutions x' and x''.

Consider the function $\Psi(\rho, x)$ obtained from Equation (1) by setting $y = y_0$. It implicitly defines a function $\rho = \psi(x)$ and, by construction, $\phi(x') = \phi(x'') = \rho_0$.

In order to prove the theorem, we prove that ψ is concave for any x between x' and x'' and, then, the value of ρ is greater than ρ_0.

Consider the first derivative of ψ w.r.t. x. Since ψ is implicitly defined, according to Dini's Theorem,

$$\frac{d\psi}{dx} = -\frac{\partial\Psi/\partial x}{\partial\Psi/\partial\rho} = -\frac{-\rho^2 + 2\rho x - y_0}{3\rho^2 - 2\rho x y_0 + (x^2 + y_0^2 - 1)}.$$

In order to study the growth of the function, we have to solve the following system:

$$\begin{cases} -\dfrac{-\rho^2 y_0 + 2\rho x - y_0}{3\rho^2 - 2\rho x y_0 + (x^2 + y_0^2 - 1)} \geq 0 & (2) \\ \rho^3 - \rho^2 x y_0 + \rho(x^2 + y_0^2 - 1) - x y_0 = 0 & (3) \end{cases}$$

First of all, note that the denominator of Equation (2) is always greater than 0.

Indeed, if $3\rho^2 - 2\rho x y_0 + (x^2 + y_0^2 - 1) < 0$ then $x^2 + y_0^2 - 1 = 2\rho x y_0 - 3\rho^2 - \epsilon$ for some $\epsilon > 0$. But this value make unsatisfiable Equation (3), since

$$\rho^3 - \rho^2 x y_0 + \rho(2\rho x y_0 - 3\rho^2 - \epsilon) - x y_0 =$$
$$= (-2\rho^3 - x y_0(1 - \rho^2) - \epsilon) < 0$$

for any $\epsilon > 0$ and $\rho \leq 1$.

Thus, by considering just numerator, Equation (2) is satisfied for any

$$x \leq \frac{(\rho^2 + 1) y_0}{2\rho} \tag{4}$$

and the derivative is 0 for $x = \overline{x} = \frac{(\rho^2+1)y_0}{2\rho}$

By solving Equation (3) w.r.t. x we obtain solutions

$$
\begin{aligned}
x' &= \frac{1}{2\rho}\left(y_0(\rho^2+1) - \sqrt{y_o^2(\rho^2-1)^2 - 4\rho^2(\rho^2-1)}\right) \\
x'' &= \frac{1}{2\rho}\left(y_0(\rho^2+1) + \sqrt{y_o^2(\rho^2-1)^2 - 4\rho^2(\rho^2-1)}\right)
\end{aligned} \tag{5}
$$

By coupling Equation (4) with Equations (5), we obtain that x' is smaller than $\overline{x}$, x'' is larger than $\overline{x}$ and the function increases before $\overline{x}$ and decreases after $\overline{x}$. Thus, x is a maximum and all the values of x in $[x', x'']$ are such that $\psi(x) > \rho_0$, qde.

Let call t'_i and t''_i the points obtained by solving Equation (1) when dealing with the expression level of sample t on gene a_i, and t'_j and t''_j the points we get for sample t with respect to its expression on gene a_j; then, the probabilities P_i^+ and P_j^+ can be rewritten as:

$$
\begin{aligned}
P_i^+ &= Pr(\widehat{X}_i \le t''_i) - Pr(\widehat{X}_i \le t'_i) = \Phi(t''_i) - \Phi(t'_i) \\
P_j^+ &= Pr(\widehat{X}_j \le t''_j) - Pr(\widehat{X}_j \le t'_j) = \Phi(t''_j) - \Phi(t'_j),
\end{aligned}
$$

where $\Phi(\cdot)$ denotes the cumulative distribution function of the standard normal distribution.

1.2 Upper Bound

Let $\mathbf{N}$ be a set of *WIGA-networks* partitioned into two sub-set $\mathbf{N}_1$ and $\mathbf{N}_2$ and let $s(\mathcal{P}, \mathbf{N}_1)$ be the incidence of a pattern $\mathcal{P}$ in $\mathbf{N}_1$. The upper bound on the discriminative power that can be obtained by extending $\mathcal{P}$ can be computed assuming that the incidence of the patterns obtained from $\mathcal{P}$ remains unchanged in $\mathbf{N}_1$ and becomes zero in the other set $\mathbf{N}_2$. Therefore, you need to evaluate the discriminative power $pow(\mathcal{P})$ choosing $\widehat{s}(\mathcal{P}, \mathbf{N}_2) = 0$. In this scenario, the entropy terms which appears in the information entropy formula of def. 10 in the main paper become:

$$
\begin{aligned}
H(\mathbf{N}^{\mathcal{P}}) =& 0 \\
H(\mathbf{N}^{\overline{\mathcal{P}}}) =& - q_2 \log q_2 - (1 - q_2) \log(1 - q_2)
\end{aligned}
$$

Thus, $H(\mathbf{N}^{\overline{\mathcal{P}}})$ is unchanged while $H(\mathbf{N}^{\mathcal{P}})$ becomes 0 as $q1 = 1$.

As already pointed out in the main paper, the definition of information entropy is not symmetric; indeed if you are intended to detect patterns characterizing $\mathbf{N}_2$ but not $\mathbf{N}_1$ the upper bound can be computed reasoning in the same way but and evaluating $pow(\mathcal{P})$ when $s(\mathcal{P}, \mathbf{N}_1) = 0$.

1.3 Function PatternMine

Function PATTERNMINE($\mathcal{P}$)

```
Input: Current analysed pattern P
Output: Set of discriminative patterns res obtained from P
res ← ∅;
Lp ← rankExtension(P);
foreach P' in Lp do
    if isDiscriminative(P') then
        res ← res ∪ P';
    if isExtensible(P') then
        return res ∪ PatternMine(P');
    else
        break;
return res;
```

2 Results on Synthetic Data

The proposed approach has been evaluated in a simulation scenario built on synthetic data. In particular, a pair of datasets have been randomly generated, each containing 256 samples described by 25 attributes (i.e., the gene expression values simulated for each sample, respectively), for 10 times. Then, two groups of attributes, one of size 2 and the other of size 3, have been chosen to simulate high co-expression between genes in each group in one of the population against the other one, and check the ability of our method to detect possible discriminative patterns, injected this way, and correctly involving genes in the two modified groups. In more detail, to simulate high co-expression between genes in the two groups, their corresponding entries in one population have been substituted by data coming from a multivariate normal distribution with a number of dimensions equal to the size of the associated group. By properly choosing the covariance matrix associated to the normal distribution, we are able to ensure that the selected genes result to be correlated each other. Such a correlation will lead to a strong association, in terms of both strength and relevance, of the selected genes in the corresponding WIGA networks associated with each sample.

WIGA networks have been constructed for each of the 10 runs, with each node associated to one out of the 25 attributes, and the proposed approach applied accordingly. As for the values injected to simulate high co-expression, the first group of attributes correspond to nodes labelled by 0 and 1 in the networks, while the second group to nodes 2, 3 and 4, respectively.

The approach has returned from 17 to 20 discriminative patterns, for the 10 different runs. In all runs, 4 patterns involving the two groups of nodes with injected values have been returned: a linear pattern involving the two nodes 0 and 1, and other three linear patterns involving 2, 3 and 4, respectively. We call *injected patterns* such 4 patterns, for short. It is worth pointing out that non-linear patterns involving nodes 2, 3 and 4 have been discarded, since their discriminative power is lower than the linear returned ones. Table 1 summarizes the structures of the best scoring discriminative patterns detected over the 10 runs, together with the mean of their discriminative power, and their position spanning within the rank based on the discriminative power of all obtained patterns. As an example, the position of pattern $0 - 1$ in the rank spans from 1 to 4 over the 10 runs. The 4 injected patterns are always the best scoring ones, and they alternate each other across the 4 first positions of the rank in the different runs. Therefore, this first experiment has been successful in showing the effectiveness of our approach.

Pattern Structure	***Mean ± Standard Deviation***	**Position spanning**
$0 - 1$	0.0571 ± 0.0199	1/4
$4 - 2 - 3$	0.0646 ± 0.008	2
$2 - 3 - 4$	0.0598 ± 0.014	1/3
$2 - 4 - 3$	0.0686 ± 0.0101	2/3

Table 1: Injected patterns features.

Another experiment has been performed on synthetic data to test if our technique is robust to noise, as described below. Given a contamination level c (expressed as a percentage of the number of samples in the datasets), c samples of the two populations are randomly exchanged and the ability of the proposed approach in finding out the injected patterns is evaluated in the new induced scenario. Table 2 shows the results for different values of the contamination level c. Here, again, the mean of the discriminative power values obtained over the 10 runs is considered. Note that, although the mean of the discriminative power values is lower than the one detected without noise, the method is always capable of detecting the two-size injected pattern; even for the three-size patterns, at least one of them is detected despite the contamination level, showing that the method can be considered robust to noise.

To provide also a quantitative evaluation of the accuracy of the proposed approach, the AUC over the rank obtained by sorting patterns based on (the mean of) their discriminative power values is shown in Table 3, for each contamination level. Without noise, the injected patterns are always at the top positions in the rank, therefore in that case the AUC is always equal to 1. The mean of the AUC values, obtained over the 10 runs and for each percentage of noise, spans from 0.805 to 0.989, thus confirming the robustness of our method. Moreover, this latter test shows also that, possible false positives returned by the proposed approach, are not among those patterns scoring the highest values of discriminative power.

Contamination Level	*Mean ± Standard Deviation*	**Best rank position**	**Worst rank position**
**0 − 1**			
5%	0.0329 ± 0.0131	1	4
10%	0.0258 ± 0.008	1	5
15%	0.022 ± 0.0093	1	7
20%	0.045 ± 0.0069	1	4
**4 − 2 − 3**			
5%	Not detected		
10%	0.0209 ± 0.0037	2	5
15%	0.0209 ± 0.0037	2	5
20%	0.0157 ± 0.0	4	4
**2 − 3 − 4**			
5%	0.0279 ± 0.0	2	2
10%	0.0276 ± 0.0	2	2
15%	Not detected		
20%	Not detected		
**2 − 4 − 3**			
5%	Not detected		
10%	0.029 ± 0.0	1	1
15%	0.0363 ± 0.0	2	2
20%	0.0133 ± 0.0001	11	13

Table 2: Robustness analysis.

Contamination Level	*Mean of AUC values over* 10 *runs* ± *Standard Deviation*
5%	0.989 ± 0.019
10%	0.955 ± 0.074
15%	0.881 ± 0.101
20%	0.805 ± 0.164

Table 3: AUC computation.

3 Results on Real Data

Microarray chips are designed to analyse a fixed number of probes, i.e. single helix DNA fragments, to quantify the expression of specific mRNA sequences complementary to them. The number of probes depends on the platform used for the experiments (see *#Probes* column in tab. 4).

Data used for our experiments have been normalized using Robust Multi-array Average method Irizarry *et al.* (2003) implemented in Bioconductor using default settings. After that, probe ids have been mapped on genes names in order to remove those data not corresponding to any gene. Furthermore, as some genes can exist that are mapped on more than one probes, we have handled them by taking into account the median among the expression levels measured for the target gene by each probe. Table 4 summarizes the main characteristics of the datasets we use for ouu experiments. All dataset are accessible at NCBI GEO database Edgar *et al.* (2002) through the GEO Series accession number reported in the table.

In this section some more details are provided about the results we get by running our algorithm on the datasets presented above. Table 5 reports the main characteristics of the result-sets referred to each dataset.

3.1 Functional Enrichment Analysis

As an example, we report in Figure 1 one of the connected components for the global view of Pancreas Cancer (Healthy).

	GEO Series accession number			
	**GSE68907** Singh *et al.* (2002)	**GSE15471** Idichi *et al.* (2017), Badea *et al.* (2008)	**GSE65801** Li *et al.* (2015)	**GSE13355** Nair *et al.* (2009), Ding *et al.* (2010)
Disease	Prostate Cancer	Pancreas Cancer	Gastric Cancer	Psoriasis
Platform	GPL8300	GPL570	GPL14550	GPL570
*#**Healthy***	50	39	32	64
*#**Unhealthy***	52	39	32	58
*#**Probes***	12625	54675	42545	54675
*#**Not mapped***	1152	12734	12712	12734
*#**Duplicates***	2032	10757	6234	10757
*#**Genes***	8596	20192	21754	20192

Table 4: Description of the dataset used in the experiments. Note that, for each dataset the table reports the number of probes, the one not mapped to any gene and the duplicates (one or more times) that have to be subtracted to get the number of genes used for the analysis.

	**N. of patterns**		**Maximum size**	
	Healthy	Unhealthy	Healthy	Unhealthy
**Prostate Cancer**	286	654	11	9
**Pancreas Cancer**	370	416	8	8
**Gastric Cancer**	323	309	6	6
**Psoriasis**	479	501	6	2

Table 5: Summary of the extracted discriminative patterns features per dataset.

3.2 Shape of the Resulting Patterns

To go in depth with the structural characteristics of the patters detected by our method we consider, for each dataset, the top-12 patterns which score the highest values of commonness. In Table 6 only those of Prostate Cancer are plotted, since for the other diseases similar results have been obtained. The pictures highlight that in most cases the genes which take part in the patterns are organised to form non-linear structures, i.e. the interactions that make them discriminative are generally no-trivial.

Prostate Cancer - Healthy

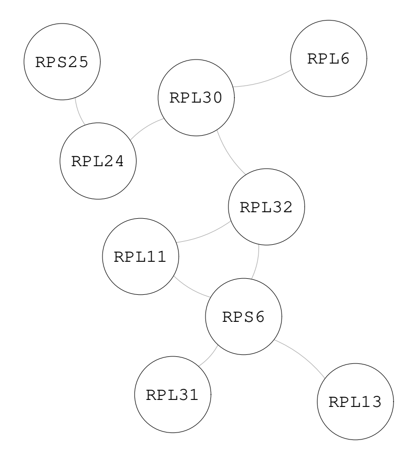

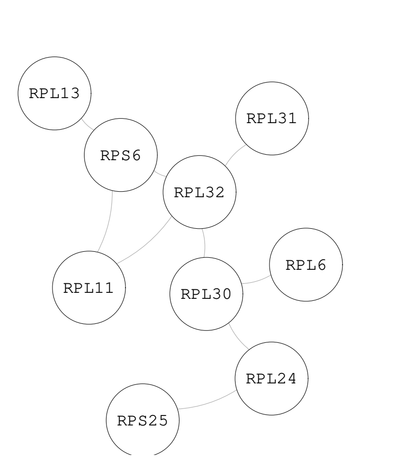

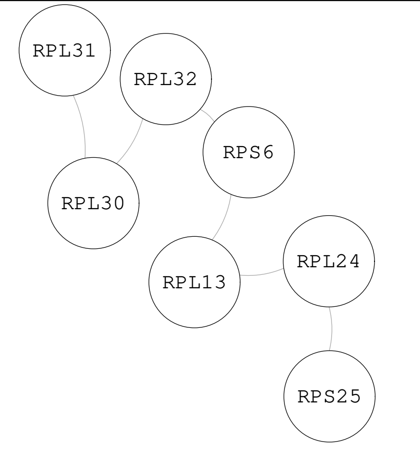

continue to next page

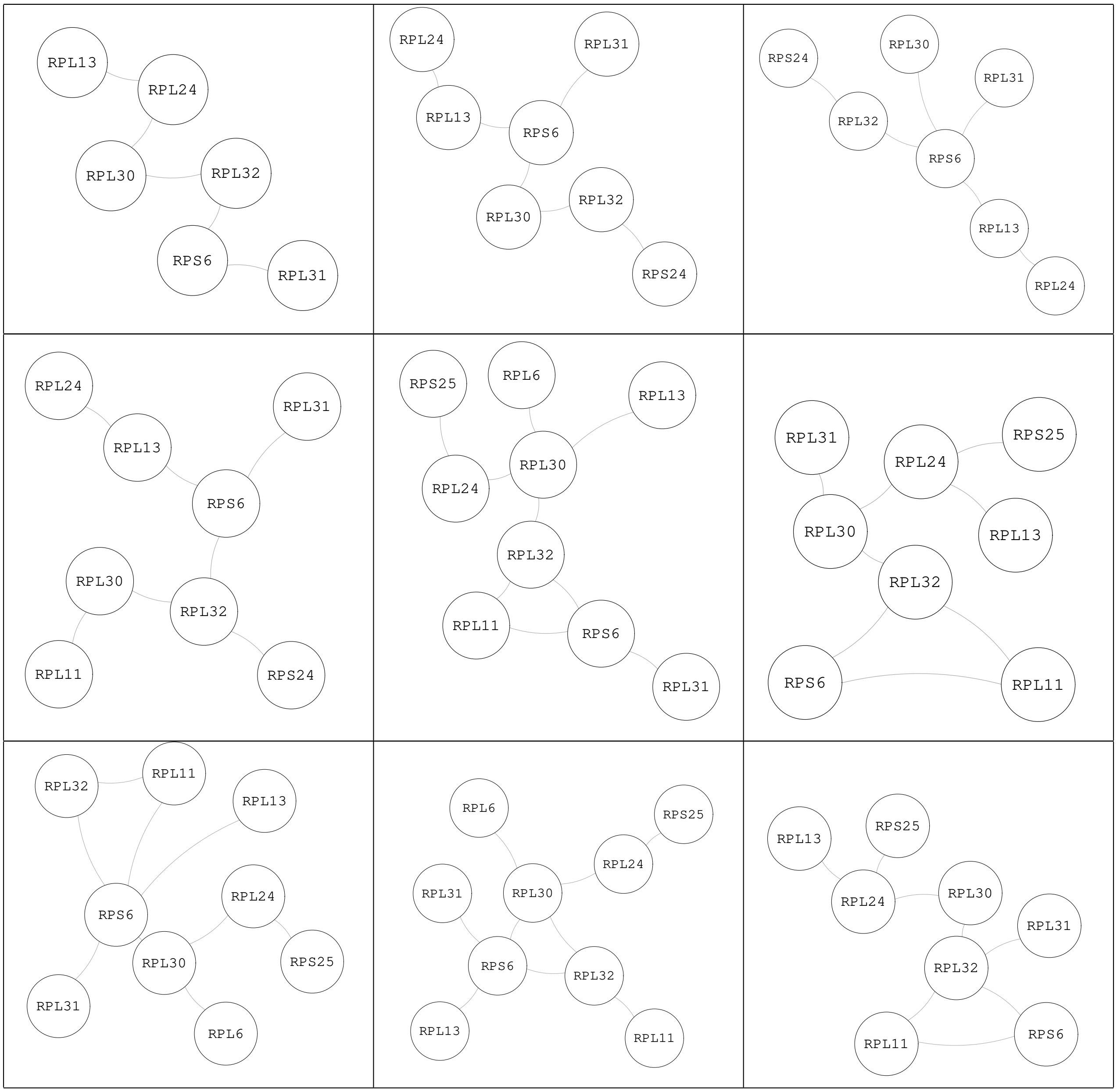

Prostate Cancer - Unhealthy

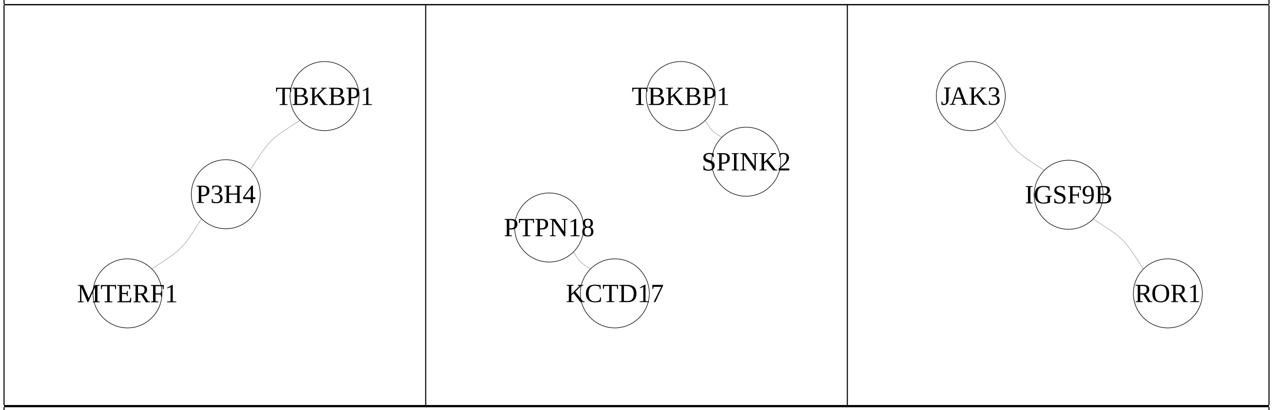

continue to next page

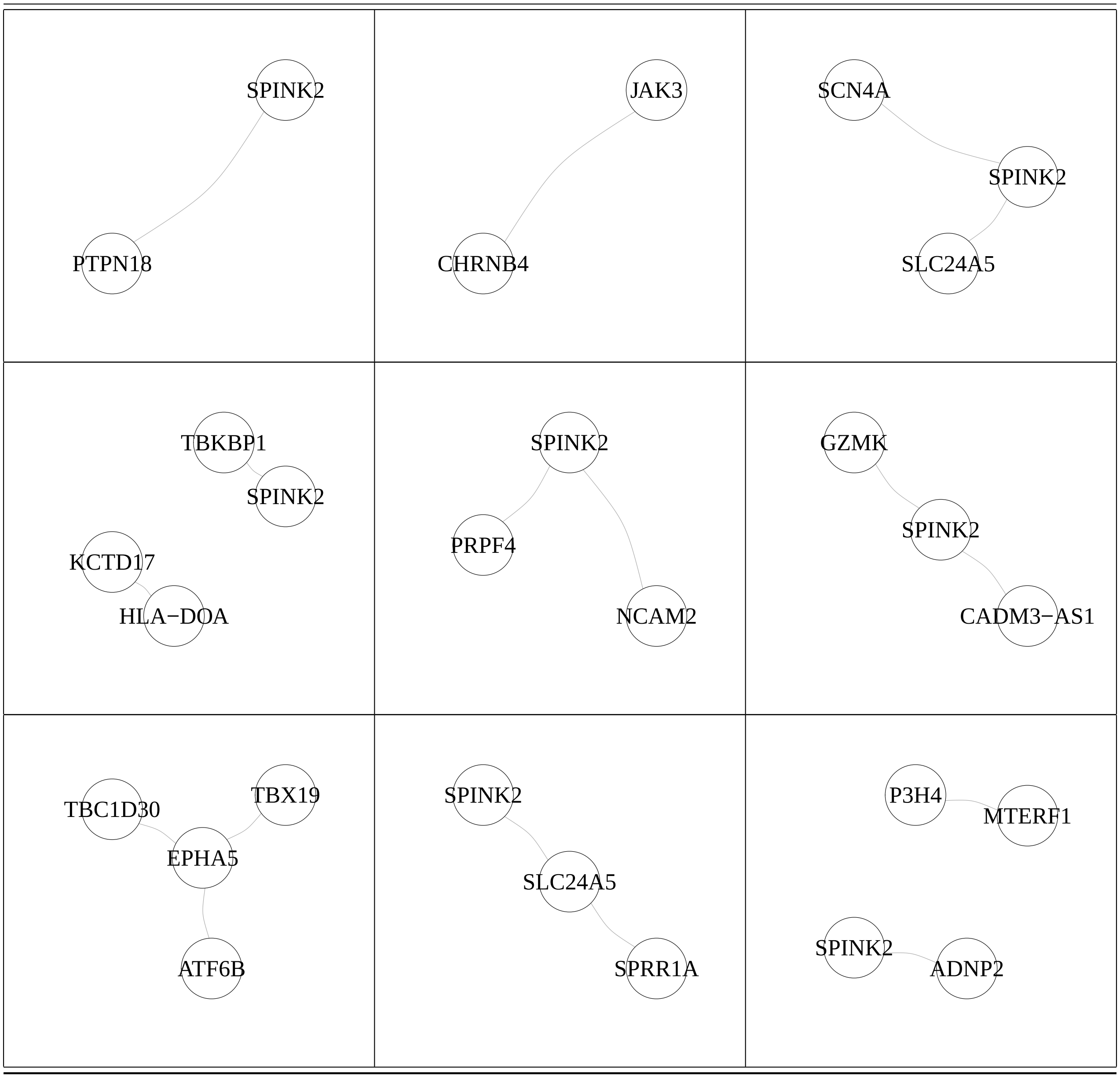

Table 6: Top-12 patterns getting the best commonness values for the Prostate Cancer dataset.

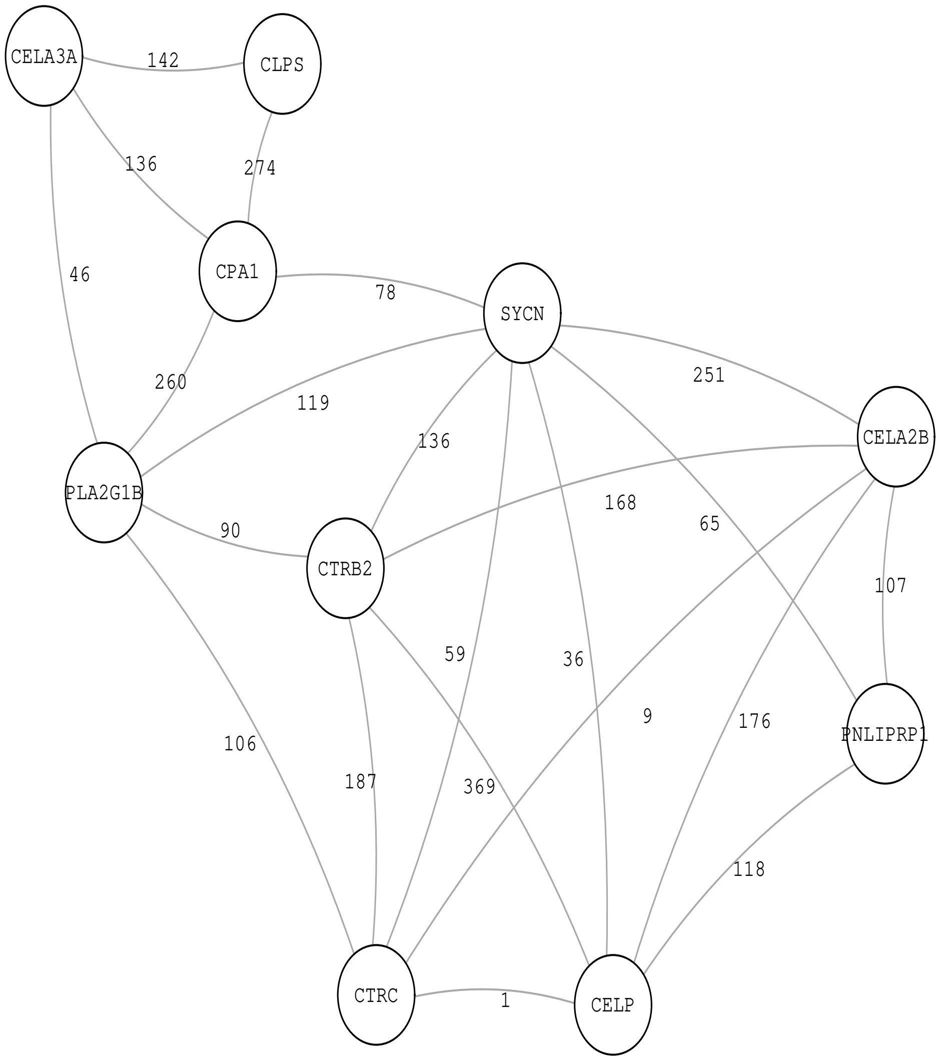

Figure 1: One of the connected components for the global view of Pancreas Cancer (Healthy).

3.3 Frequency of Occurrence Analysis

Patters detected by our method are often built based on a small subset of genes. Tables 7 - 10 list, for each dataset, the top-10 most frequent single genes, pairs, triples and quadruples detected in the result-set .

Prostate Cancer

Healthy	Unhealthy
RPL13	SPINK2
RPS6	AMELX
RPL32	HSPB1
RPL30	RPL10A
RPL31	RPL11
RPL11	RPS18
RPL24	RPS4X
RPL37	POU3F1
RPL27	JAK3
RPS25	SCN4A

Pancreas Cancer

Healthy	Unhealthy
CELA2B	REG1A
CELA3A	REG1B
CELP	CTRB2
CLPS	CPB1
CPA1	PLA2G1B
CTRB2	CELA3B
CTRC	CELA3A
PLA2G1B	CPA2
PNLIPRP1	PRSS3P2
SYCN	CEL

Gastric Cancer

Healthy	Unhealthy
RTL1	OR9K2
C1orf129	OR6S1
SERPINA13	BTG4
ADAM6	LOC285766
PSG3	KRTAP4-3
LOC401445	LGALS13
CXorf66	IFLTD1
F9	OR2T10
TAS2R1	OR11H4
CFHR5	SPINLW1

Psoriasis

Healthy	Unhealthy
KRT35	RNF148
KRTAP3-3	ACMSD
KRTAP4-1	APLNR
KRTAP1-3	C11orf86
KRTAP4-3	C8orf46
KRT86	CELP
PNPLA2	CES1P1
CALCOCO1	CETN1
LRP10	CHRDL2
KDM5D	CHST7

Table 7: First ten most frequent genes in the extracted discriminative patterns for all datasets.

Prostate Cancer

Healthy	Unhealthy
RPS6, RPL32	RPL11, HSPB1
RPL32, RPL30	RPS18, RPL10A
RPL32, RPL11	RPS4X, RPL11
RPS6, RPL13	RPL11, POU3F1
RPS6, RPL31	RPL10A, AMELX
RPL37, RPL13	P3H4, MTERF1
RPL30, RPL27	RPL10A, MARCKS
RPS25, RPL24	SPINK2, SCN4A
RPS6, RPL11	RPL11, AMELX
RPL30, RPL24	SPINK2, SLC24A5

Pancreas Cancer

Healthy	Unhealthy
CELP, CELA2B	CELA3A, CEL
CLPS, CELA3A	CELA3B, CEL
CPA1, CELA3A	CELA3B, CELA3A
CPA1, CLPS	CPA1, CELA3A
CTRB2, CELA2B	CPA1, CELA3B
CTRB2, CELP	CPA2, CELA3A
CTRC, CELA2B	CPA2, CELA3B
CTRC, CELP	CPA2, CPA1
CTRC, CTRB2	CPB1, CEL
PLA2G1B,CELA3A	CPB1, CELA3A

Gastric Cancer

Healthy	Unhealthy
RTL1, C1orf129	OR9K2, BTG4
PSG3, C1orf129	OR9K2, OR6S1
SERPINA13, RTL1	OR9K2, LOC285766
RTL1, PSG3	OR6S1, KRTAP4-3
SERPINA13, ADAM6	OR6S1, OR2T10
SERPINA13, C1orf129	OR6S1, LGALS13
F9, C1orf129	OR9K2, IFLTD1
RTL1, ADAM6	OR9K2, LGALS13
TAS2R1, RTL1	LGALS13, IFLTD1
C1orf129, ADAM6	LOC285766, IFLTD1

Psoriasis

Healthy	Unhealthy
KRTAP4-3, KRTAP3-3	ABI3, ABCC11
KRTAP1-3, KRT35	ATF4, ARRDC
KRTAP4-1, KRTAP3-3	ATL2, ARL14EP
KRTAP4-1, KRTAP1-3	ATP13A1, ASB17
KRTAP3-3, KRTAP1-3	ATP1A3, ACTL8
KDM5D, EIF1AY	AURKAIP1, ATP13A4AS1
KRTAP4-7, KRT86	BDNF, APOBEC3D
PNPLA2, LRP10	C1orf131, BRMS1
KRTAP9-4, KRTAP4-1	CA5A, ASB5
KRTAP1-3, KRT86	CAPN12, C5orf46

Table 8: First ten most frequent pairs of genes in the extracted discriminative patterns for all datasets.

Prostate Cancer

Healthy	Unhealthy
NACA, RPL24, RPL30	HSPB1, RPL11, RPS4X
RPL11, RPL19, RPS6	HSPB1, POU3F1, RPL11
RPL13, RPL19, RPS6	POU3F1, RPL11, RPS4X
RPL13, RPL24, RPL27	AMELX, RPL10A, RPS18
RPL13, RPL24, RPL32	MARCKS, RPL10A, RPS18
RPL13, RPL24, RPL35	AMELX, RPL11, RPS4X
RPL13, RPL27, SRP14	AMELX, HSPB1, RPL11
RPL13, RPL31, RPL37	AMELX, MARCKS, RPL10A
RPL13, RPL32, RPL35	AMELX, POU3F1, RPL11
RPL24, RPL32, RPS25	AMELX, RPL10A, RPL11

Pancreas Cancer

Healthy	Unhealthy
CELA2B, CELP, CTRB2	CTRB2, REG1B, REG3A
CELA2B, CTRB2, SYCN	CPB1, CTRB2, REG3A
CELA3A, CLPS, CPA1	CPB1, CTRB2, REG1B
CLPS, CPA1, PLA2G1B	CELA3B, CPA2, PLA2G1B
CELP, CTRB2, CTRC	CELA3B, PLA2G1B, PRSS3P2
CELP, CTRB2, SYCN	CPB1, REG1A, REG1B
CELA2B, CELP, SYCN	CPB1, CTRB2, REG1A
CPA1, PLA2G1B,SYCN	CPB1, REG1A, REG3A
CELP, CTRB2, PNLIPRP1	CELA3B, CPB1, REG1A
CELA3A, CPA1, PLA2G1B	CELA3A, REG1A, REG3A

Gastric Cancer

Healthy	Unhealthy
C1orf129,PSG3,RTL1	BTG4,OR6S1,OR9K2
C1orf129,RTL1,SERPINA13	LGALS13,OR6S1,OR9K2
C1orf129,CXorf66,PSG3	BTG4,LOC285766,OR9K2
C1orf129,PSG3,SERPINA13	BTG4,IFLTD1,OR9K2
ADAM6,C1orf129,SERPINA13	IFLTD1,LOC285766,OR9K2
PSG3,RTL1,SERPINA13	BTG4,LGALS13,OR9K2
ADAM6,RTL1,SERPINA13	KRTAP4-3,OR6S1,OR9K2
C1orf129,F9,RTL1	IFLTD1,LGALS13,LOC285766
ADAM6,CXorf66,SERPINA13	IFLTD1,OR6S1,OR9K2
C1orf129,RTL1,TAS2R1	OR2T10,OR6S1,OR9K2

Psoriasis

Healthy	Unhealthy
KRTAP1-3, KRTAP3-3, KRTAP4-1	ERF, ISOC2, QTRT1
KRTAP3-3, KRTAP4-1, KRTAP4-3	IRF7, ISG15, RSAD2
KRT35, KRTAP1-3, KRTAP3-3	LRRN4CL, PLAC9, TGFBR3
KRTAP1-3, KRTAP3-3, KRTAP4-3	NPRL3, SDHC, ZBTB45
KRT35, KRTAP1-3, KRTAP4-1	SLC35A2, TAPBP, TFE3
KRT35, KRT86, KRTAP1-3	-
KRT35, KRTAP3-3, KRTAP4-3	-
KRTAP3-3, KRTAP4-1, KRTAP9-4	-
EIF1AY, KDM5D, UHMK1	-
KRT86, KRTAP1-3, KRTAP3-3	-

Table 9: First ten most frequent ternes of genes in the extracted discriminative patterns for all datasets.

Prostate Cancer

Healthy	Unhealthy
RPL30, RPL31, RPL32, RPS6	HSPB1, POU3F1, RPL11, RPS4X
RPL11, RPL30, RPL32, RPS6	AMELX, HSPB1, RPL11, RPS4X
RPL11, RPL31, RPL32, RPS6	AMELX, RPL10A, RPL11, RPS4X
RPL11, RPL13, RPL32, RPS6	AMELX, POU3F1, RPL11, RPS4X
RPL13, RPL31, RPL32, RPS6	AMELX, HSPB1, POU3F1, RPL11
RPL13, RPL30, RPL32, RPS6	AMELX, MARCKS, RPL10A, RPS18
RPL27, RPL30, RPL32, RPS6	AMELX, HSPB1, RPL10A, RPL11
RPL11, RPL30, RPL31, RPL32	AMELX, RPL10A, RPL11, RPS18
RPL13, RPL30, RPL32, RPL37	AMELX, POU3F1, RPL10A, RPL11
RPL11, RPL24, RPL30, RPL32	AMELX, RPL10A, RPL19, RPS18

Pancreas Cancer

Healthy	Unhealthy
CELA2B,CELP,CTRB2,SYCN	CPB1,CTRB2,REG1B,REG3A
CELA3A,CLPS,CPA1,PLA2G1B	CPB1,CTRB2,REG1A,REG3A
CELA2B,CELP,CTRB2,PNLIPRP1	CPB1,CTRB2,REG1A,REG1B
CELA2B,CELP,CTRB2,CTRC	CEL,CPB1,CTRB2,REG3A
CELA2B,CTRB2,CTRC,SYCN	CELA3A,CELA3B,CPB1,REG1A
CLPS,CPA1,PLA2G1B,SYCN	CELA3B,CPB1,CTRB2,REG3A
CELP,CTRB2,CTRC,SYCN	CELA3A,CPB1,CTRB2,REG3A
CELP,CTRB2,CTRC,PLA2G1B	CELA3A,CELA3B,CPA2,PLA2G1B
CELA2B,CELP,PNLIPRP1,SYCN	CEL,CPB1,CTRB2,REG1B
CTRB2,CTRC,PLA2G1B,SYCN	CELA3A,CELA3B,REG1A,REG3A

Gastric Cancer

Healthy	Unhealthy
C1orf129, PSG3, RTL1, SERPINA13	BTG4, LGALS13, OR6S1, OR9K2
ADAM6, C1orf129, RTL1, SERPINA13	IFLTD1, LOC285766, OR9K2, RBM46
ADAM6, C1orf129, PSG3, SERPINA13	IFLTD1, LGALS13, OR6S1, OR9K2
C1orf129, CXorf66, PSG3, RTL1	BTG4, IFLTD1, OR6S1, OR9K2
C1orf129, CXorf66, PSG3, SERPINA13	KRTAP4-3, LGALS13, OR6S1, OR9K2
ADAM6, C1orf129, CXorf66, PSG3	BTG4, OR6S1, OR9K2, TRPC5
ADAM6, C1orf129, CXorf66, SERPINA13	OR11H4, OR14C36, OR6S1, OR9K2
ADAM6, C1orf129, PSG3, RTL1	BTG4, IFLTD1, LOC285766, OR9K2
C1orf129, CXorf66, RTL1, SERPINA13	IFLTD1, LGALS13, LOC285766, OR6S1
CXorf66, PSG3, RTL1, SERPINA13	IFLTD1, LOC285766, OR6S1, OR9K2

Psoriasis

Healthy	Unhealthy
KRTAP1-3, KRTAP3-3, KRTAP4-1, KRTAP4-3	-
KRT35, KRTAP1-3, KRTAP3-3, KRTAP4-1	-
KRT35, KRTAP1-3, KRTAP3-3, KRTAP4-3	-
KRTAP1-3, KRTAP3-3, KRTAP4-1, KRTAP9-4	-
KRTAP3-3, KRTAP4-1, KRTAP4-3, KRTAP9-4	-
KRT35, KRTAP3-3, KRTAP4-1, KRTAP4-3	-
KRT35, KRT86, KRTAP1-3, KRTAP3-3	-
KRT86, KRTAP1-3, KRTAP3-3, KRTAP4-3	-
EIF1AY, KDM5D, UHMK1, USP9Y	-
KRT86, KRTAP3-3, KRTAP4-3, KRTAP4-7	-

Table 10: First ten most frequent quadruples of genes in the extracted discriminative patterns for all datasets.

3.4 Hub Genes Identification via PPI Network Analysis

We investigated the role of the genes involved in the extracted patterns by taking into account a human protein-protein interaction (PPI) network, built by downloading data from IntACT (Orchard *et al.*, 2014). We focus on those genes having at least 10 connection within the network, which are significantly larger in the two cases of Prostate Cancer and Psoriasis (see table 11). More in details, table 12 reports, for each dataset and for each of the two analysed scenarios, Healthy and Unhealthy, the top-25 genes (if available), ordered by the number of connection they have within the PPI network, which is also reported in the table next to the gene name.

	**Prostate Cancer**	**Pancreas Cancer**	**Gastric Cancer**	**Psoriasis**
Healthy	19	0	2	258
Unhealthy	215	2	7	223

Table 11: Number of hub genes for each dataset and each analysed case.

Prostate Cancer

**Healthy**		**Unhealthy**	
RPS6	84	HSPB1	349
RPL11	77	EEF1A1	296
RPL6	71	PABPC1	155
RPL24	65	DHX9	138
RPS18	64	RIF1	127
RPL31	61	MET	107
RPS7	50	RPS3	106
RPL35	46	PKM	104
RPL19	43	CIAO1	103
RPS23	41	AMOT	96
SRP14	41	HSPA1L	95
RPS24	39	RPS4X	93
RPL13	38	IKZF1	93
NACA	35	NCOR2	92
RPL27	34	CASP8	86
RPL32	23	DLG4	85
RPL30	23	EIF2AK2	85
RPL37	15	RPS6	84
RPS25	13	PSMA7	81
		RPLP0	80
		DDX21	80
		RPL11	77
		MAPT	76
		SLC25A6	75
		RBL1	74

Psoriasis

**Healthy**		**Unhealthy**	
RELA	314	AGO1	196
PPP2R1A	184	CALCOCO2	172
FLNA	148	AKT1	147
IGSF8	148	PRPF31	129
BAG6	139	CACYBP	110
CD247	138	IRAK1	106
FBXW11	135	ATF4	88
AP2M1	127	GFI1B	86
KRTAP4-12	120	LUC7L2	84
ATN1	114	CDC23	83
KRTAP5-9	110	KRT18	83
RAD23A	109	MAPK9	78
EHMT2	108	PPP2R1B	75
PKM	104	POLR2E	74
FTSJ1	100	CCNA2	71
CACNA1A	97	MAP3K7	71
AMOT	96	SMARCD1	71
ARAF	91	CCAR2	70
BYSL	86	DDX6	69
PRPF8	84	STX11	69
GNB2	83	TCEA2	69
AMOTL2	80	AIMP2	66
SAT1	80	SNRPA	66
EIF3E	79	MYH10	64
ACTN4	76	SNRPD2	62

Pancreas Cancer

**Healthy**		**Unhealthy**	
		FHL1	50
		KRT20	32

Gastric Cancer

**Healthy**		**Unhealthy**	
PAX2	21	IRS4	103
CDC20B	12	GMCL1P1	21
		PAX2	21
		TRPC5	17
		OPRK1	11
		BOLL	10

Table 12: Top-25 hub genes and their corresponding degrees in the IntACT PPI network.

3.5 Comparison Against a Standard Approach

Here the proposed approach is compared against a standard one for studying gene expression variation between two populations, that is, searching for genes differentially expressed in the two populations, without considering co-expression between genes. The main idea guiding this set of experiments is that, genes involved in co-expression patterns, are not necessarily expected to be found as relevant, if considered alone. Instead, part of them come out in the analysis only within discriminative patterns involving other significantly co-expressed genes, characterizing a population in contrast to the other

one. Therefore, the proposed approach is useful to identify genes with important roles in the onset and progress of diseases, that otherwise would remain unseen.

The comparison has been performed over all the datasets associated to the four considered diseases. In particular, for each gene, the expression value in all the available samples has been considered, and the t-test has been computed to compare its expression trend in the two populations. The p-value resulting from the test has been used to quantify the statistical significance of the differences in the expression trend over the two sub-populations, and genes have been sorted accordingly.

Then, for each dataset, genes involved in at least the 20% of the returned patterns have been considered, identifying their position within the p-value-based ranking. Such genes are listed in Tables 13-16, together with the scored p-value (second column), their position in the p-value ranking divided by the whole number of genes in the dataset (third column) and the value of log fold change (fourth column). Highest the value in the third column, lowest the gene position in the ranking. For three out of the four considered datasets, as expected, the statistical significance of many (although not all) considered genes is poor, according to the t-test, when they are considered alone. Interestingly, for the Prostate Cancer dataset, genes coding for ribosomal proteins and frequent in patterns characterizing the healthy population, come out with high significance also in the differentially expressed search. Also for the log fold change values, which divergence to the zero value represents to what extent the gene expression value differs between the two population, it is evident that the genes considered in the tables would not be considered as extremely significant. This emerges also in the Volcano plots represented in Figure 2, which combine a measure of statistical significance (the p-value of the t-test) with the magnitude of the change in expression level of the genes between the two populations involved in the analysis (fold change). In more detail, the negative logarithm of the p-value has been reported on the y-axis, such that data points with low p-values, i.e., the most significant ones, are shown at the top of the plot. On the other hand, those points that are both at the top of the plot and far to either the left- or right-hand sides, correspond to large magnitude fold changes as well as to high statistical significance.

The green and yellow squares on the plots denote the areas where the genes reported in Tables 13-16 are located, based on their p-value and log fold change. Interestingly, they are not in the positions that are worth of attention for a traditional differentially expressed analysis, thus confirming that it would be difficult to identify them through a standard analysis.

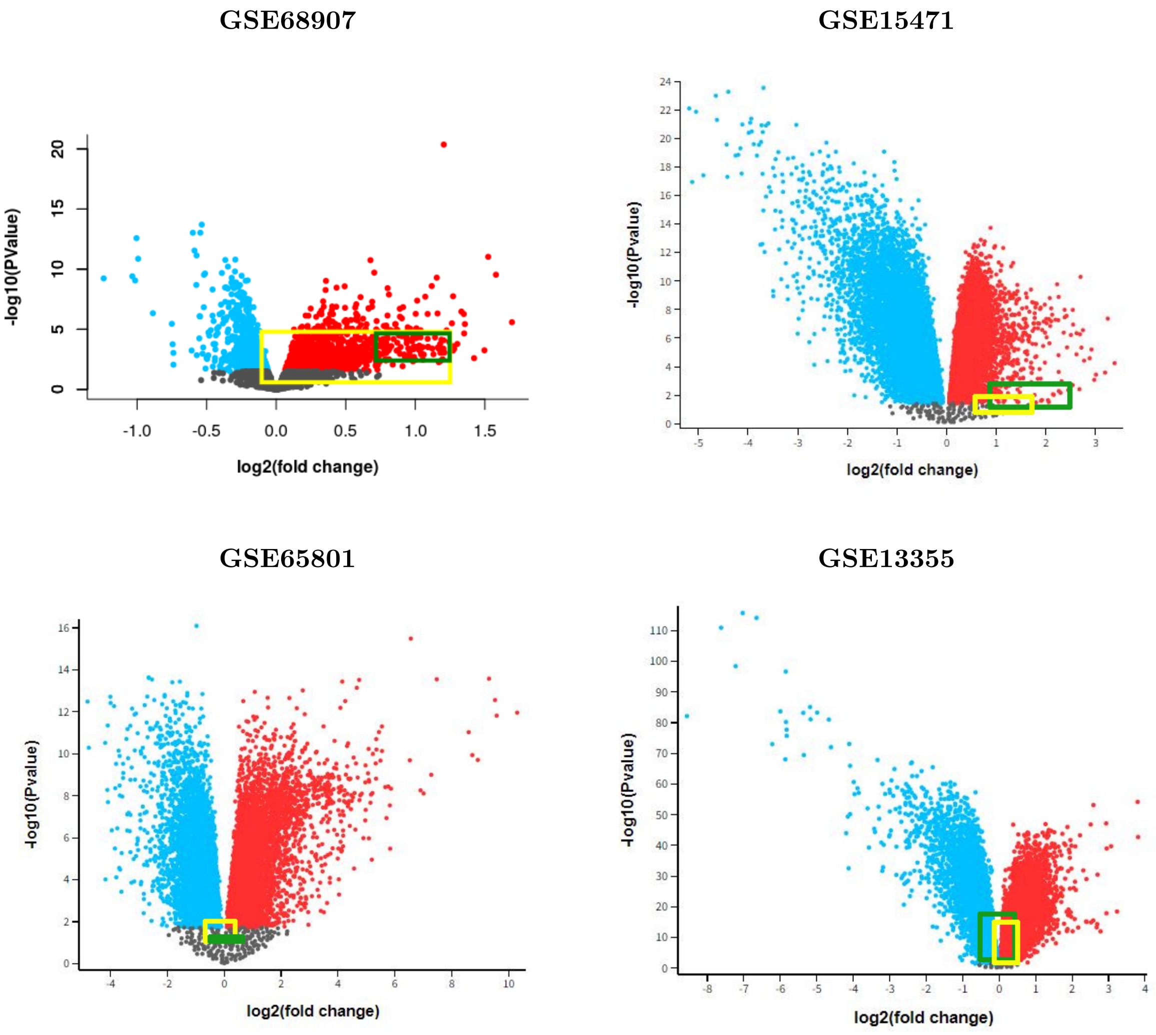

Figure 2: Volcano Plots. The plots combine a measure of statistical significance (the p-value of the t-test) with the magnitude of the change in expression level of the genes involved in the analysis (fold change). The red dots refer to the genes that are more expressed in the healthy samples, while the blue ones refer to genes more expressed in unhealthy samples. The green and yellow squares delimitate the area on the plot where the genes which are more frequent in our discriminative patterns are located, for healthy and unhealthy samples, respectively.

Prostate Cancer			
Gene	***P-value***	***Position Percentage***	***logFC***
**Healthy Samples**			
RPS18	2.04E-04	6.03	1.30
RPL6	1.37E-04	5.35	1.24
RPS25	3.55E-04	6.97	0.81
RPL27	5.51E-04	7.98	0.99
RPL37	1.06E-04	4.99	0.94
RPL24	3,61E-04	7.03	0.90
RPL11	1.08E-03	9.80	1.04
RPL31	3.48E-03	14.66	1.20
RPL30	2.10E-03	12.15	0.97
RPL32	1.47E-03	10.80	1.06
RPS6	1.05E-03	9.77	1.18
RPL13	2.17E-04	6.10	0.75
**Unhealthy samples**			
SPINK2	1.70E-01	62.13	-0.15
AMELX	8.57E-02	7.48	-0.08
HSPB1	7.74E-01	64.16	-0.074
RPL10A	1.41E-03	64.17	1.13
RPL11	1.08E-03	93.83	1.04
RPS18	2.04E-04	28.58	1.30
RPS4X	2.50E-03	48.34	0.93
POU3F1	2.39E-01	41.66	-0.05
JAK3	4.53E-02	2.13	-0.12
SCN4A	1.38E-01	41.02	-0.10

Table 13: Genes frequent in the discriminative patterns for the considered dataset (first column), scored p-value resulting from the t-test (second column), position in the p-value rank divided by the whole number of genes in the dataset (third column).

Pancreas Cancer			
Gene	***P-value***	***Position Percentage***	***logFC***
**Healthy Samples**			
CELP	9.63e-03	64.87	2.16
CTRB2	2.88e-01	86.32	0.94
CELA2B	4.52e-03	60.91	2.45
CPA1	1.59e-01	81.84	1.36
PLA2G1B	1.18e-01	79.73	1.51
CLPS	5.30e-02	74.69	1.72
CTRC	5.09e-02	74.44	1.64
CELA3A	1.72e-01	82.41	1.19
PNLIPRP1	2.01e-03	56.98	2.54
SYCN	3.87e-03	60.20	2.67
**Unhealthy samples**			
PRSS3P2	7.55e-02	76.86	1.12
CPA2	3.34e-02	71.85	1.80
CELA3A	1.72e-01	82.41	1.19
CELA3B	1.37e-01	80.84	1.27
PLA2G1B	1.18e-01	79.73	1.51
CPB1	5.10e-01	91.58	0.56
CTRB2	2.88e-01	86.32	0.94
REG1B	5.43e-01	92.22	0.51
REG1A	1.35e-01	80.73	1.15

Table 14: Genes frequent in the discriminative patterns for the considered dataset (first column), scored p-value resulting from the t-test (second column), position in the p-value rank divided by the whole number of genes in the dataset (third column).

Gastric Cancer			
Gene	***P-value***	***Position Percentage***	***logFC***
**Healthy Samples**			
PSG3	1.25e-01	55.29	-0.21
ADAM6	3.79e-01	73.27	0.089
SERPINA13	6.51e-01	86.10	0.076
C1orf129	4.95e-01	79.06	0.044
RTL1	3.21e-01	69.87	-0.099
LOC401445	4.47e-01	69.21	0.152
CXorf66	4.60e-01	69.88	0.079
F9	7.80e-01	87.35	0.037
TAS2R1	2.92e-01	59.68	-0.16
CFHR5	6.57e-01	80.74	-0.052
**Unhealthy samples**			
IFLTD1	2.92e-01	68.13	0.085
KRTAP4-3	3.07e-01	69.06	-0.27
LGALS13	5.77e-01	82.92	0.034
LOC285766	2.25e-01	63.60	-0.271
BTG4	3.94e-01	74.07	-0.059
OR6S1	1.70e-01	59.31	-0.422
OR9K2	2.96e-01	68.40	-0.148
LGALS13	8.91e-01	93.65	0.0344
IFLTD1	4.29e-01	68.13	0.0846
OR2T10	6.01e-02	39.04	-0.543

Table 15: Genes frequent in the discriminative patterns for the considered dataset (first column), scored p-value resulting from the t-test (second column), position in the p-value rank divided by the whole number of genes in the dataset (third column).

Psoriasis			
Gene	***P-value***	***Position Percentage***	***logFC***
**Healthy Samples**			
KRTAP1-3	4.46e-01	87.05	0.26
KRTAP4-3	2.13e-01	78.78	0.36
KRT35	5.53e-02	68.13	0.49
KRTAP3-3	7.18e-01	93.82	0.14
KRTAP4-1	8.93e-01	97.73	-0.013
KRT86	5.82e-01	90.67	0.14
PNPLA2	2.16e-01	78.94	-0.063
CALCOCO1	1.25e-11	19.23	0.41
LRP10	3.85e-18	24.88	-0.34
KDM5D	6.44e-02	69.16	0.12
**Unhealthy samples**			
RNF148	9.52e-01	98.94	1.35e-03
ACMSD	7.54e-01	94.61	6.58e-03
APLNR	3.42e-03	53.27	1.93e-01
C11orf86	1.37e-03	49.78	-1.32e-01
C8orf46	1.43e-01	75.15	-3.97e-02
CELP	1.01e-02	58.17	-1.66e-01
CES1P1	1.35e-06	32.57	-2.86e-01
CETN1	1.54e-03	50.22	-9.98e-02
CHRDL2	1.42e-04	42.33	-1.61e-01
CHST7	4.95e-12	18.45	4.87e-01

Table 16: Genes frequent in the discriminative patterns for the considered dataset (first column), scored p-value resulting from the t-test (second column), position in the p-value rank divided by the whole number of genes in the dataset (third column).

References

Badea, L., Herlea, V., Dima, S. O., Dumitrascu, T., Popescu, I., *et al.* (2008). Combined gene expression analysis of whole-tissue and microdissected pancreatic ductal adenocarcinoma identifies genes specifically overexpressed in tumor epithelia-the authors reported a combined gene expression analysis of whole-tissue and microdissected pancreatic ductal adenocarcinoma identifies genes specifically overexpressed in tumor epithelia. *Hepato-gastroenterology*, **55**(88), 2016.

Ding, J., Gudjonsson, J. E., Liang, L., Stuart, P. E., Li, Y., Chen, W., Weichenthal, M., Ellinghaus, E., Franke, A., Cookson, W., *et al.* (2010). Gene expression in skin and lymphoblastoid cells: Refined statistical method reveals extensive overlap in cis-eqtl signals. *The American Journal of Human Genetics*, **87**(6), 779–789.

Edgar, R., Domrachev, M., and Lash, A. E. (2002). Gene expression omnibus: Ncbi gene expression and hybridization array data repository. *Nucleic acids research*, **30**(1), 207–210.

Idichi, T., Seki, N., Kurahara, H., Yonemori, K., Osako, Y., Arai, T., Okato, A., Kita, Y., Arigami, T., Mataki, Y., *et al.* (2017). Regulation of actin-binding protein anln by antitumor mir-217 inhibits cancer cell aggressiveness in pancreatic ductal adenocarcinoma. *Oncotarget*, **8**(32), 53180.

Irizarry, R. A., Hobbs, B., Collin, F., Beazer-Barclay, Y. D., Antonellis, K. J., Scherf, U., and Speed, T. P. (2003). Exploration, normalization, and summaries of high density oligonucleotide array probe level data. *Biostatistics*, **4**(2), 249–264.

Li, H., Yu, B., Li, J., Su, L., Yan, M., Zhang, J., Li, C., Zhu, Z., and Liu, B. (2015). Characterization of differentially expressed genes involved in pathways associated with gastric cancer. *PloS one*, **10**(4), e0125013.

Nair, R. P., Duffin, K. C., Helms, C., Ding, J., Stuart, P. E., Goldgar, D., Gudjonsson, J. E., Li, Y., Tejasvi, T., Feng, B.-J., *et al.* (2009). Genome-wide scan reveals association of psoriasis with il-23 and nf-κb pathways. *Nature genetics*, **41**(2), 199–204.

Orchard, S., Ammari, M., Aranda, B., *et al.* (2014). The MIntAct project—IntAct as a common curation platform for 11 molecular interaction databases. *Nucleic Acids Res*, **42**(D1), D358–D363.

Singh, D., Febbo, P. G., Ross, K., Jackson, D. G., Manola, J., Ladd, C., Tamayo, P., Renshaw, A. A., D'Amico, A. V., Richie, J. P., *et al.* (2002). Gene expression correlates of clinical prostate cancer behavior. *Cancer cell*, **1**(2), 203–209.
